# The EBNA3 Family of Epstein-Barr Virus Nuclear Proteins Associates with the USP46/USP12 Deubiquitination Complexes to Regulate Lymphoblastoid Cell Line Growth

**DOI:** 10.1371/journal.ppat.1004822

**Published:** 2015-04-09

**Authors:** Makoto Ohashi, Amy M. Holthaus, Michael A. Calderwood, Chiou-Yan Lai, Bryan Krastins, David Sarracino, Eric Johannsen

**Affiliations:** 1 Departments of Medicine and Oncology (McArdle Laboratory for Cancer Research), University of Wisconsin, Madison, Wisconsin, United States of America; 2 Infectious Disease Division, Department of Medicine, Brigham and Women’s Hospital and Harvard Medical School, Boston, Massachusetts, United States of America; 3 Biomarker Research Initiatives in Mass Spectrometry (BRIMS), Thermo Fisher Scientific, Cambridge, Massachusetts, United States of America; Duke University Medical Center, UNITED STATES

## Abstract

The Epstein-Barr virus (EBV) nuclear proteins EBNA3A, EBNA3B, and EBNA3C interact with the cell DNA binding protein RBPJ and regulate cell and viral genes. Repression of the CDKN2A tumor suppressor gene products p16^INK4A^ and p14^ARF^ by EBNA3A and EBNA3C is critical for EBV mediated transformation of resting B lymphocytes into immortalized lymphoblastoid cell lines (LCLs). To define the composition of endogenous EBNA3 protein complexes, we generated lymphoblastoid cell lines (LCLs) expressing flag-HA tagged EBNA3A, EBNA3B, or EBNA3C and used tandem affinity purification to isolate each EBNA3 complex. Our results demonstrated that each EBNA3 protein forms a distinct complex with RBPJ. Mass-spectrometry revealed that the EBNA3A and EBNA3B complexes also contained the deubquitylation complex consisting of WDR48, WDR20, and USP46 (or its paralog USP12) and that EBNA3C complexes contained WDR48. Immunoprecipitation confirmed that EBNA3A, EBNA3B, and EBNA3C association with the USP46 complex. Using chromatin immunoprecipitation, we demonstrate that WDR48 and USP46 are recruited to the p14^ARF^ promoter in an EBNA3C dependent manner. Mapping studies were consistent with WDR48 being the primary mediator of EBNA3 association with the DUB complex. By ChIP assay, WDR48 was recruited to the p14^ARF^ promoter in an EBNA3C dependent manner. Importantly, WDR48 associated with EBNA3A and EBNA3C domains that are critical for LCL growth, suggesting a role for USP46/USP12 in EBV induced growth transformation.

## Author Summary

Epstein-Barr virus (EBV) is a gammaherpesvirus implicated in the pathogenesis of multiple malignancies, including Burkitt lymphoma, Hodgkin lymphoma, post-transplant lymphoproliferative disease (PTLD), nasopharyngeal carcinoma, and gastric carcinoma. EBV infection of resting B-lymphocytes drives them to proliferate as lymphoblastoid cell lines (LCLs), an in vitro model of PTLD. LCLs express a limited EBV gene repertoire, including six nuclear proteins (EBNA1, 2, 3A, 3B, 3C, and LP), three integral membrane proteins (LMP1, 2A, and 2B), and more than 30 micro RNAs. EBNA2 and the EBNA3 proteins are transcription factors that regulate viral and cell gene expression through the cell DNA binding protein RBPJ. In this study, we established LCLs transformed by recombinant EBV genomes in which a Flag-HA epitope tag is fused in-frame to the C-terminus of EBNA3A, EBNA3B or EBNA3C. Using these LCLs, we purified endogenous EBNA3 complexes and identified the USP46 deubiquitinating enzyme (DUB) and its associated chaperones WDR48 and WDR20 as EBNA3 binding proteins. We find that EBNA3s interact primarily with the WDR48 protein and that loss of WDR48 interaction with EBNA3A or EBNA3C impairs LCL growth. This study represents the first characterization of EBNA3 complexes from LCLs and implicates the USP46 DUB complex in EBNA3 mediated gene regulation.

## Introduction

Epstein-Barr Virus (EBV) is a herpesvirus that establishes lifelong asymptomatic infection in up to 95% of the human population [1]. In vitro, EBV infection of resting B lymphocytes drives them to proliferate as lymphoblastoid cell lines (LCLs) [2,3]. The EBV genome resides in LCLs as a non-integrated episome and expresses a limited gene repertoire called latency III, which includes genes encoding six nuclear proteins (EBNA1, 2, 3A, 3B, 3C, and LP), three integral membrane proteins (LMP1, 2A, and 2B), and more than 30 micro RNAs (miRs) [1]. Latency III driven B lymphocyte proliferation in vivo is normally controlled by a vigorous cytotoxic T cell response [4]. In the absence of an effective immune response or in collaboration with various environmental or genetic co-factors, EBV latent infection can result in malignancies, including Burkitt and Hodgkin lymphomas, post-transplant lymphoproliferative disease (PTLD), as well as nasopharyngeal and gastric carcinomas [1].

Extensive genetic and biochemical data support the model that EBV latency III gene expression usurps growth and survival signaling pathways in B lymphocytes normally triggered by antigen recognition and CD4+ T cell co-stimulation [1,5]. LMP1 expression results in constitutive NF-kB activation that is essential for LCL outgrowth and survival. The ability of LMP1 to self-associate allows it to activate, in a ligand independent manner, molecules that transduce signals from receptors in the TNF superfamily [6,7,8,9,10]. The other two latent membrane proteins, LMP2A and LMP2B, are not required for LCL transformation in vitro [11]. The ability of LMP2A to engage B cell receptor signaling molecules may be important for maintaining viral latency or for the growth and survival of EBV infected cells in vivo [12,13]. EBNA2 is an acidic transactivator that is targeted to promoters through an interaction with the RBPJ DNA binding protein, a component of the Notch signaling pathway [14]. EBNA2 and its co-activator EBNALP are the first genes expressed during EBV latent infection and result in upregulation of promoters including c-myc, EBV LMP1, LMP2A, and EBNA essential for latency III transformation [15,16]. Global analysis of EBNA2 and RBPJ binding in LCLs has implicated EBF1 and other B cell transcription factors as pioneering factors for EBNA2 binding of promoters and enhancers [17]. In contrast, the role of the EBNA3 proteins in LCL transformation is less clearly defined.

The EBNA3 protein family is defined by a ~300 aa region of homology in their N-termini; there are no known homologs outside of EBV and the closely related primate lymphocryptoviruses. EBNA3A, EBNA3B, and EBNA3C share a common exon structure consisting of a short 5’ exon and a longer 3’ exon arranged in a tandem array that likely arose from triplication of a single ancestral EBNA3 gene [1]. Reverse genetic analyses have demonstrated that EBNA3C is essential for LCL transformation, while EBNA3B is dispensable [18,19]. The requirement for EBNA3A is probably intermediate. EBNA3A truncation or conditional inactivation abrogated transformation in multiple studies. However, LCLs have been generated under appropriate conditions, using feeder cells, with an EBV genome deleted for EBNA3A [20]. The most convincing evidence of the unique requirement for EBNA3A and EBNA3C derives from LCLs in which either EBNA3A or EBNA3C has been rendered conditional by fusion to a mutant estrogen hormone binding domain [21,22,23,24,25]. In this system, LCL growth arrest induced by EBNA3A inactivation could be rescued only by exogenous EBNA3A expression, but not by expression of additional EBNA3B or EBNA3C [22,23]. Similarly, EBNA3C inactivation results in termination of LCL growth that can only be restored by EBNA3C [21,24,25].

Cell cycle effects of the EBNA3 proteins, particularly EBNA3C, have been documented in many other systems. EBNA3C can overcome serum deprivation and disrupt the G1 checkpoint in REFs, NIH3T3, and U2OS cells [26]. Additionally EBNA3C overexpression can disrupt mitotic spindle checkpoints and produce aneuploidy [26,27]. EBNA3A and EBNA3C can cooperate with HRAS in classical transformation assays [28]. Multiple mechanisms of EBNA3C mediated effects have been suggested, including inhibition of accumulation of the CDK inhibitors p27^KIP1^ and p16^INK4A^, Rb degradation via the SCF ubiquitin ligase, c-myc stabilization, and binding to, and inactivation of cyclinA-CDK complexes [29,30,31]. In LCLs, the growth effects of EBNA3A and EBNA3C appear to be primarily due to suppression of the CDKN2A gene products p16^INK4A^ and p14^ARF^ [32]. Conditional inactivation of either EBNA3A or EBNA3C results in p16^INK4A^ and p14^ARF^ accumulation and cessation of growth. Moreover, siRNA knockdown of both gene products restores growth despite EBNA3A or EBNA3C inactivation. EBNA3A and EBNA3C effects appear to be at the level of CDKN2A transcription as changes in protein levels are accompanied by concomitant increases in mRNA and a substantial reduction of the repressive H3K27me3 modification at the CDKN2A promoter [32]. Furthermore, p16^INK4A^ null B lymphocytes can be transformed into LCLs in the absence of functional EBNA3C protein [33].

Although the mechanism by which EBNA3A and EBNA3C cooperatively suppress CDKN2A is unknown, gene co-regulation by the EBNA3 proteins appears to be frequent. In LCLs, 52 out of 287 genes reported as EBNA3A regulated were found to also be regulated by EBNA3C [34]. In Burkitt lymphoma cells, EBNA3A and EBNA3C are both required for suppression of BIM, a pro-apoptotic Bcl-2 family member [35]. Genome-wide analysis of EBNA3A, EBNA3B, and EBNA3C effects in BL31 cells infected with recombinant EBV genomes [36], suggested that about half of the cell genes differentially expressed as a result of deletion of one EBNA3 ORF are similarly affected by deletion of at least one of the other EBNA3s. In that study, overlap among genes regulated by EBNA3B and EBNA3C was particularly extensive [36].

A large number of interacting proteins have been suggested to be important for EBNA3 activities. Of these, RBPJ is the best established as a mediator of transcriptional and LCL growth promoting effects [37,38,39,40]. RBPJ is bound by the conserved N-terminal EBNA3 domain, which unlike Notch and EBNA2, does not interact with the RBPJ’s beta-trefoil domain. Instead, the EBNA3s bind to the N-terminal rel-homology domain (NTD) of RBPJ [37,41]. Although biochemical assays suggested that the EBNA3-NTD interaction could inhibit RBPJ DNA binding, genome-wide co-localization between EBNA3 proteins and RBPJ has been demonstrated by ChIP-seq [42,43]. Genetic analyses have demonstrated that interaction of both EBNA3A and EBNA3C with RBPJ is essential for CDKN2A promoter repression and maintenance of LCL growth [32,33]. A second cell protein important for CDKN2A regulation is CtBP1, which interacts with the C-terminal regions of EBNA3A and EBNA3C [44,45,46]. Mutation of the CtBP1 binding sites in EBNA3A and EBNA3C impairs their ability to support LCL growth. By contrast, RBPJ binding mutants are completely defective in maintenance of LCL growth [21,24].

The strength of evidence supporting a role for other interacting proteins in mediating EBNA3 growth effects varies considerably. Although significant progress has been made in mapping the EBNA3 domains critical for LCL growth, for most interacting proteins, mutations within the EBNA3 proteins that selectively disrupt their binding have yet to be identified. In parallel with efforts to correlate ongoing genetic analysis of the EBNA3 proteins with interacting protein binding, we set out to devise a means of purifying endogenous EBNA3 complexes from LCLs and to determine their protein constituents. To that end, recombinant EBV genomes in which DNA encoding a flag-HA (F-HA) epitope is inserted in-frame to the C-terminus of the EBNA3A, EBNA3B, or EBNA3C ORF were constructed. These genomes were used to transform primary B-lymphocytes into three cell lines: EBNA3A-F-HA, EBNA3B-F-HA, and EBNA3C-F-HA LCLs. Using tandem affinity purification and LC/MS/MS, we characterized the protein composition of endogenous EBNA3A, EBNA3B, and EBNA3C complexes in these LCLs.

Here we show that each EBNA3 protein is associated with the USP46 and USP12 deubiquitylase (DUB) complexes, and that the domains of the EBNA3A and EBNA3C proteins that bind these DUBs are important for maintenance of LCL growth. In the presence of EBNA3 proteins, RBPJ and the USP46/USP12 enzymes become associated and, when purified, these EBNA3 containing complexes exhibit DUB activity. Using CRIPSR/Cas9 we provide evidence that USP46 is essential in 721 LCLs, but dispensable in 293T cells. Further, using chromatin immunoprecipitation we demonstrate increased binding of WDR48 to the p14^ARF^ promoter in the presence of functional EBNA3C protein. We propose a model in which EBNA3s serve as adaptor proteins between USP46/USP12 and RBPJ, recruiting these DUB complexes to chromatin to regulate transcription.

## Results

### Production and characterization of LCLs expressing epitope tagged EBNA3 proteins

In order to study endogenous EBNA3 complexes from LCLs, we generated recombinant EBV genomes in which flag and HA epitope tags are fused in-frame with the carboxyl-terminus of EBNA3A, EBNA3B, or EBNA3C, using a previously described EBV BACmid [47]. These recombinant EBV genomes were used to transform B lymphocytes into LCLs, designated EBNA3A-F-HA LCL, EBNA3B-F-HA LCL, and EBNA3C-F-HA LCL, respectively, and collectively referred to as the EBNA3-F-HA LCLs. Additionally, a control a wild-type LCL was created using the unmodified EBV BACmid as the transforming genome. Western blotting of the three EBNA3-F-HA and wild-type LCLs revealed that RBPJ, EBNA1, EBNA2, EBNALP, and LMP1 levels in whole cell extracts were indistinguishable among the different cell lines (S1 Fig). The epitope tagged EBNA3 proteins were expressed at levels comparable to their wild-type counterparts and, as expected, migrated as slightly higher apparent molecular weights than the untagged proteins. Interestingly, the EBNA3B-F-HA LCL was hypomorphic for EBNA3C expression and exhibited a slower rate of growth than the other LCLs. A similar reduction in EBNA3C expression and rate of growth was previously reported in an LCL in which the EBNA3B gene was replaced by a chloramphenicol cassette [48]. Thus, the fusion of flag-HA tags to each of the EBNA3 open reading frames resulted in transformation competent EBVs that express latency proteins at levels comparable to those seen in wild-type LCLs.

### EBNA3 proteins exist in distinct complexes within LCLs

**Fig 1:**
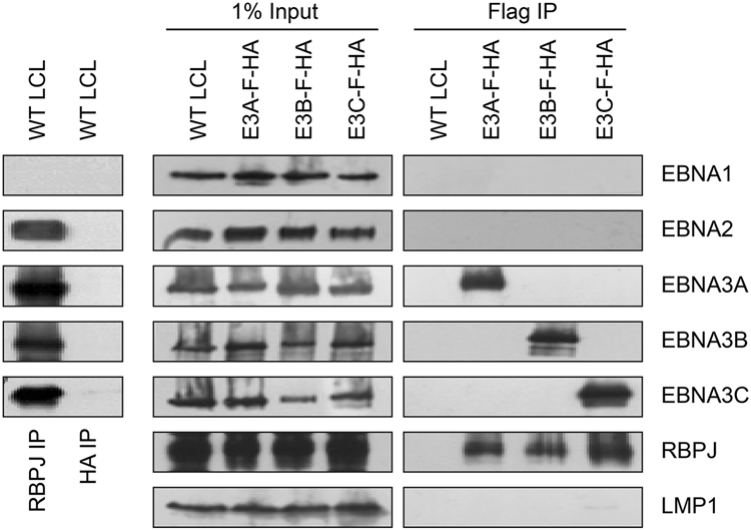
Characterization of EBNA3-F-HA LCLs, reveals that EBNA2, EBNA3A, EBNA3B, and EBNA3C form distinct RBPJ complexes. Left panel: immunoprecipitation assay for RBPJ demonstrates it is in a complex with EBNA2, EBNA3A, EBNA3B, and EBNA3C. HA immunoprecipitation serves as negative control. Right panel: Selective immunoprecipitation of EBNA3A, EBNA3B, or EBNA3C complexes from EBNA3-F-HA LCLs or WT LCL using Flag agarose. Precipitated complexes were resolved by SDS-PAGE and probed for the indicated EBV latent proteins or RBPJ. For each LCL, one percent of the input lysate is shown for comparison.

RBPJ immunoprecipitation efficiently retrieves four of the six EBV nuclear proteins (EBNA2, EBNA3A, EBNA3B, and EBNA3C) from LCL lysates (Fig 1, left panels). Although previous work had suggested that EBNA2 and EBNA3C exist in distinct complexes [49], efforts to further investigate whether EBNA3 proteins exist in distinct complexes have been hampered by varying degrees of cross-reactivity of among available EBNA3A, EBNA3B, and EBNA3C antibodies [43]. Using flag immunoprecipitation on EBNA3A-F-HA LCL lysates, we found that EBNA3A and RBPJ were efficiently precipitated, but no EBNA1, EBNA2, EBNA3B, EBNA3C or LMP1 was detectable (Fig 1, right panels). Immunoprecipitations using flag resin on EBNA3B-F-HA or EBNA3C-F-HA LCL lysates produced similar results: RBPJ and the tagged EBNA3 protein were readily detectable, but other EBV latency proteins were not. Control immunoprecipitations for HA (Fig 1, left panel) and flag (Fig 1, right panel) from wild-type LCLs, did not precipitate any detectable RBPJ, EBNA2, or EBNA3 proteins. Thus, EBNA2, EBNA3A, EBNA3B, and EBNA3C all associate with the same cell DNA binding protein, but appear to form distinct RBPJ complexes in LCLs.

### EBNA3A and EBNA3B target the USP46/USP12 complexes in LCLs

In order to identify proteins that associate with EBNA3A, EBNA3B, or EBNA3C under physiologic conditions, we purified EBNA3 complexes by tandem affinity purification (TAP) from LCLs expressing flag-HA tagged EBNA3A, EBNA3B or EBNA3C and from the wt LCL as a control. For each LCL, LC/MS/MS fingerprinting identified between 63–174 peptides of the epitope tagged EBNA3 protein and 95–148 peptides corresponding to RBPJ (Table 1). For each EBNA3-F-HA LCL, the purified protein complex contained peptides from the corresponding epitope tagged EBNA3 protein and no peptides that mapped to the other untagged EBNA3 proteins expressed in that LCL. No peptides from other EBV proteins, such as EBNA1, EBNA2, and EBNALP, were detected in any complexes. Additionally, 14 peptides corresponding to CtBP1 were detected in the EBNA3A complex, but not in the EBNA3B, EBNA3C or control TAPs (Table 1). We also detected 105, 99, and 3 total peptides corresponding to WDR48 in purified EBNA3A, EBNA3B, and EBNA3C complexes, respectively. Importantly, in the EBNA3A and EBNA3B complexes we also detected the known WDR48 associated proteins WDR20 (28 and 4 peptides, respectively), USP46 (15 and 8 peptides, respectively), and USP12 (7 and 4 peptides, respectively) (Table 1). Thus, analysis of tandem affinity purified EBNA3 complexes lends further support to model that each EBNA3 protein, while highly associated with RBPJ, exists in a distinct complex that does not contain other EBV nuclear proteins. Further, our results identify the USP46 (and USP12) deubiquitinases and their associated chaperones WDR48 and WDR20 as members of the EBNA3A and EBNA3B protein complexes in LCLs. Because USP46 and USP12 are highly homologous (~90% identity) and we could confirm a robust association with USP46 by Western blotting (discussed below), we chose to focus our subsequent attention on the USP46/WDR48/WDR20 complex.

**Table 1:** Summary of peptides identified by mass spec of tandem affinity purifications (TAP).

	Flag-HA tagged protein			
	none	EBNA3A	EBNA3B	EBNA3C
EBNA3A	0	63	0	0
EBNA3B	0	0	146	0
EBNA3C	0	0	0	174
RBPJ	0	95	148	125
CtBP1	0	14	0	0
WDR48	0	105	99	3
WDR20	0	28	4	0
USP46	0	15	8	0
USP20	0	7	4	0

Total number of peptides detected for each EBNA3 protein and the host proteins RBPJ, CtBP1, WDR48, WDR20, USP46 and USP12 in purified complexes is indicated. No peptides corresponding to any of these proteins were detected in the control TAP from wildtype LCLs.

### EBNA3C also interacts with the USP46 complex in LCLs

**Fig 2:**
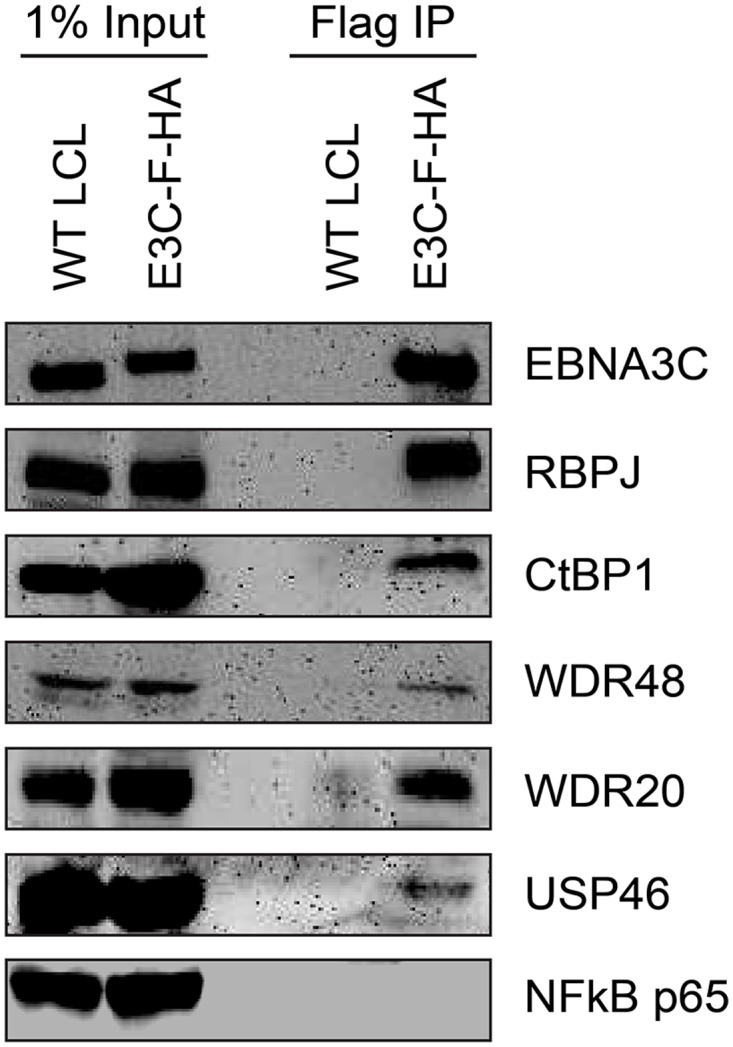
EBNA3C associates with the WDR48/USP46 complex in EBNA3C-F-HA LCLs. Immunoprecipitation assay using Flag agarose to retrieve protein complexes from EBNA3C-F-HA LCLs (E3C-F-HA) is compared to flag immunoprecipiates from untagged wildtype (WT) LCLs. One percent of total cell lysate (Input) or immunoprepicitated specimens using Flag agarose (Flag IP) were separated by SDS PAGE and probed using antibodies to EBNA3C, RBPJ, CtBP1, WDR48, WDR20, USP46, or NF-kB p65.

Given that the EBNA3 proteins share other protein binding partners (e.g., RBPJ and CtBP1) and because we had previously identified WDR48 as a cell protein that interacts with flag-EBNA3C aa365-545 [50], the small amounts of this protein detected in the EBNA3C was unexpected, particularly given the much larger amounts associated with EBNA3A and EBNA3B. We considered the possibility that WDR48 became dissociated from EBNA3C during complex purification. Therefore EBNA3C complexes were rapidly immunoprecipitated from EBNA3C-F-HA LCLs and blotted for associated proteins. Co-precipitated RBPJ was readily detected, as were members of the USP46 complex, including USP46, WDR48, and WDR20 (Fig 2). Additionally CtBP1, a known EBNA3C interacting protein [45] was detectable under these conditions even though it was not detected in the TAP-MS experiments. Thus, EBNA3C also appears to target the USP46 complex; however, this complex appears to be less stably associated with EBNA3C-F-HA than it is with either EBNA3A-F-HA or EBNA3B-F-HA.

### EBNA3-USP46 complexes are present in the nucleoplasm

**Fig 3:**
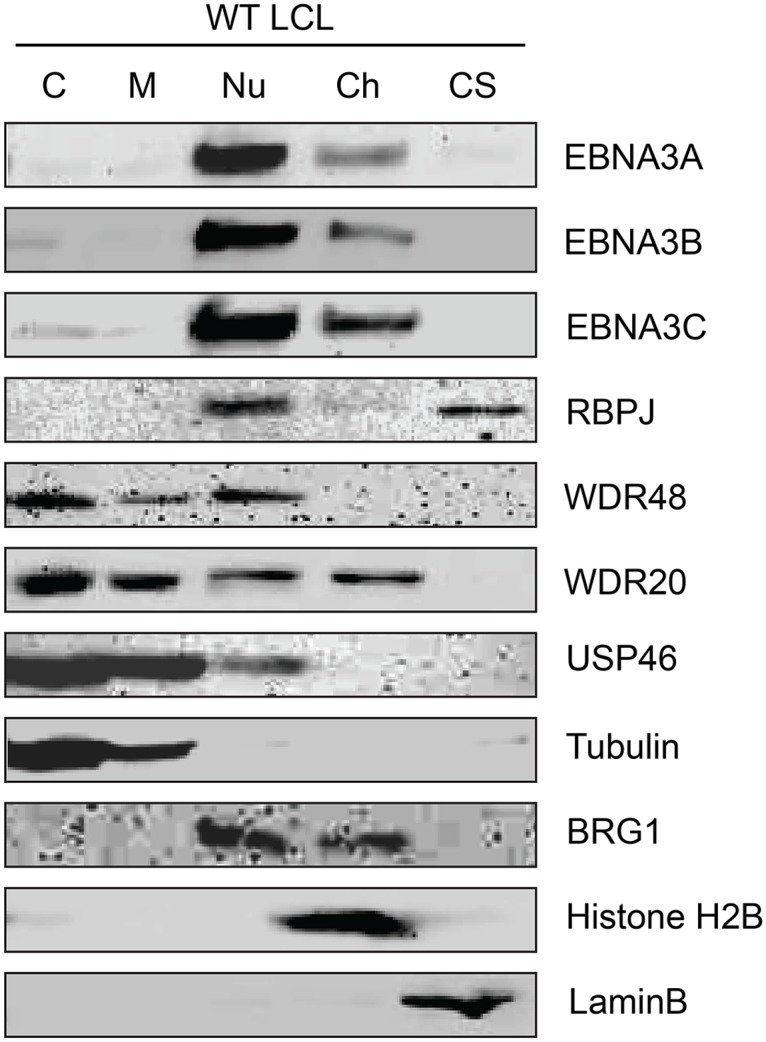
Subcellular localization of USP46 complexes in LCLs. Wild type LCLs were extracted into five subcellular fractions [cytoplasm (C), membrane (M), soluble nuclear (Nu), chromatin (Ch), cytoskeleton (CS)], resolved by SDS-PAGE and probed for EBNA3 proteins, RBPJ, and components of the USP46 complex (USP46, WDR20, and WDR48). Fraction purity was assessed by probing for tubulin, BRG1, Histone H2B, and LaminB.

Although originally described as an endosomal protein [51], WDR48 has subsequently been isolated from other cellular compartments, including as a chaperone for USP1 in the nucleus as a component of the Fanconi anemia DNA repair pathway [52]. Lehoux et al., found that USP12 and USP46 fused to red fluorescent protein were predominantly cytoplasmic in C33A cervical carcinoma cells, but were recruited to the nucleus via an interaction with the HPV E1 helicase [53]. Because our TAP lysis procedure extracted both nuclear and cytoplasmic proteins (S2 Fig), we wanted to ensure that the USP46/WDR48/WDR20 complex subcellular localization was compatible with formation of a complex with EBNA3 proteins. To this end, we fractionated LCLs into cytoplasm, membrane, nucleoplasm, chromatin, and cytoskeletal components (Fig 3). Fraction purity was monitored by immunoblotting for control proteins of known localization: alpha-tubulin (cytoplasm), BRG1 (chromatin associated), histone H2B (chromatin), and lamin B (nuclear matrix/cytoskeleton). The EBNA3 proteins were predominantly found in the nucleoplasm with a small quantity stably associated with chromatin. Notably, a significant amount of the WDR48, WDR20, and USP46 proteins were found in the nucleoplasm, with the balance being extranuclear. To more directly assess whether EBV latent gene products might affect USP46 localization, we compared fractions derived from EBV negative BL41 cells with fractions derived BL41 cells super-infected with EBV (S3 Fig). Although USP46 was present in the nucleoplasm regardless of EBV status, we consistently observed an increase in nucleoplasmic USP46 levels in EBV positive BL41 cells to varying degrees. We did not observe significant changes in the levels of nucleoplasmic WDR48 or WDR20 in response to EBV infection; however, USP46 was increased in the membrane fraction upon EBV infection (S3 Fig), similar to that seen in LCLs compared to uninfected BL41 cells. As expected, RBPJ was present in the nucleoplasm and, to a much lesser extent, the chromatin fraction in LCLs and BL41 cells. We consistently observed a portion of the cellular RBPJ in the cytoskeletal fraction of LCLs, but not in BL41 cells, regardless of EBV infection. This may reflect RBPJ association with the nuclear cytoskeleton (matrix) as has been previously reported [54]. In summary, these cell fractionation experiments are compatible with EBNA3 proteins associating with a USP46 DUB complex that resides in the nucleoplasm of B lymphocytes.

### EBNA3 proteins interact most strongly with WDR48

**Fig 4:**
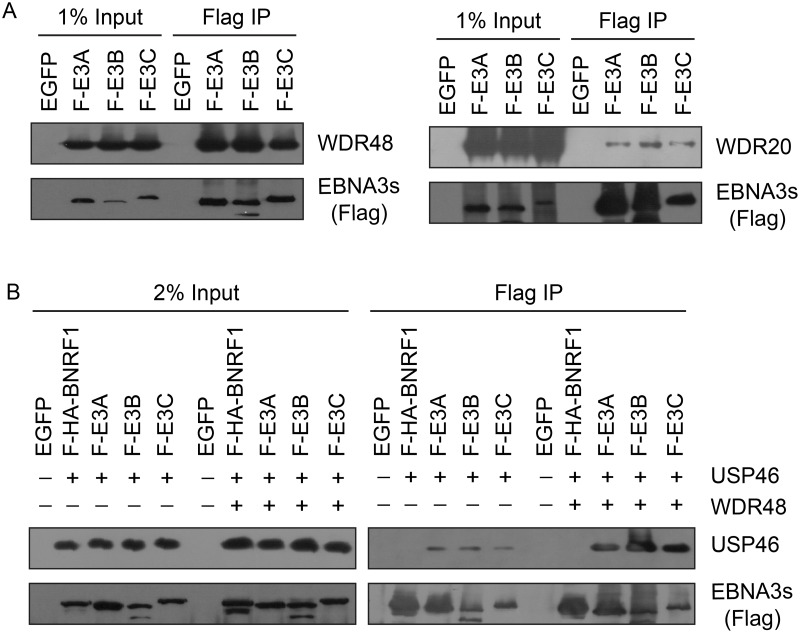
EBNA3 proteins preferentially bind the WDR48 subunit of the USP46 DUB complex. (A) Immunoprecipitation assay in 293T cells demonstrating association of flag tagged EBNA3 proteins (F-E3A, F-E3B, and F-E3C) with WDR48 (left) and WDR20 (right). (B) Immunoprecipitation assay demonstrating WDR48 cotransfection enhanced USP46 association with EBNA3s (right panel, compare lanes 3–5 with 8–10). Epitope tagged BNRF1 (F-HA-BNRF1), an EBV protein of approximately the same size as the EBNA3 is included as an additional negative control. One percent (panel A) or two percent (panel B) of the input are shown for comparison.

**Fig 5:**
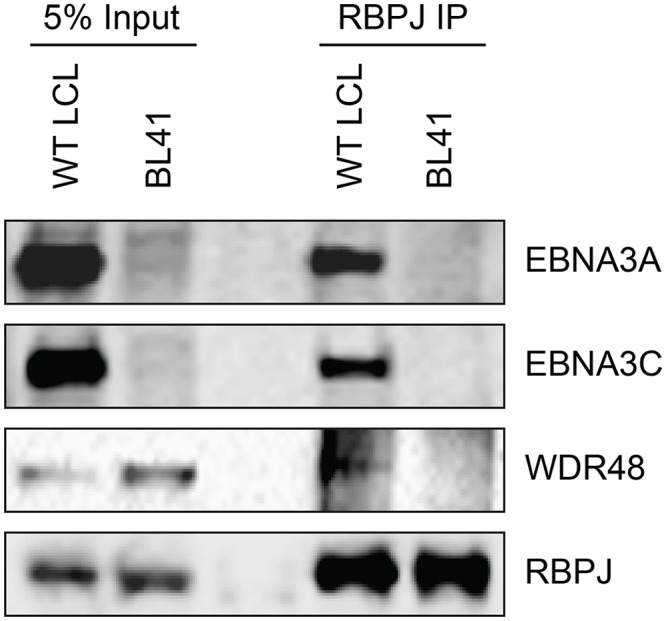
WDR48 coimmunoprecipitates with RBPJ in EBV infected cells. Co-immunoprecipitation assays comparing the association of RBPJ with WDR48 in LCLs with that observed in EBV negative BL41 cells. Cell lysates were immunoprecipitated with polyclonal RBPJ sera, separated by SDS PAGE, and probed for EBNA3A, EBNA3C, WDR48, and RBPJ (as indicated).

To further study the association of the EBNA3 proteins with members of the USP46 complex, Flag tagged EBNA3A, EBNA3B or EBNA3C was co-expressed with Xpress tagged WDR48, WDR20, or USP46. Under these conditions, each of the EBNA3 proteins interacted with WDR48, WDR20, and USP46. In each case, consistent with our LC/MS/MS data, the interaction with WDR48 was the most robust (Fig 4A and 4B). We speculated that the EBNA3 proteins may target the USP46 complex primarily via interactions with WDR48 and that, in our overexpression assay, only a small portion of the WDR20 or USP46 was complexed by endogenous WDR48. To test this, we also assessed the ability of each EBNA3 protein to co-precipitate USP46 in the presence of additional WDR48 protein (Fig 4B). This markedly increased the retrieval of USP46, suggesting a central role for WDR48 in mediating interaction with the EBNA3 proteins. In order to test whether the EBNA3 proteins could recruit this DUB complex to RBPJ complexes, we evaluated the ability of RBPJ and WDR48 to co-immunoprecipiate. In LCLs, WDR48 could be weakly detected in RBPJ immunoprecipations; however in EBV negative BL41 cells, no binding above background could be discerned (Fig 5). These results are consistent with a model in which EBNA3 proteins serve as adaptor proteins to recruit WDR48 to RPBJ in LCLs.

### WDR48 binds to EBNA3A and EBNA3C domains important for LCL proliferation

**Fig 6:**
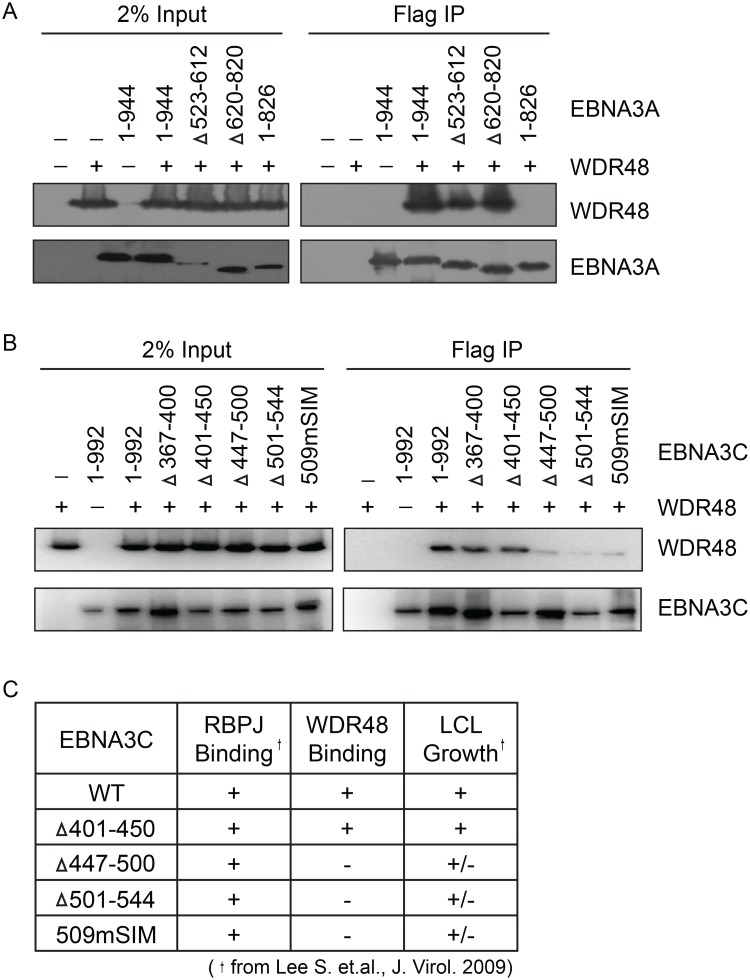
Identification of EBNA3A and EBNA3C domains that mediate WDR48 association. Immunoprecipitation assays to map WDR48 binding regions within EBNA3A (A) and EBNA3C (B). 293T cells were co-transfected with Xpress tagged WDR48 and flag tagged full length EBNA3A, EBNA3C, or the indicated EBNA3A or EBNA3C deletion mutants. Cell lysates were immunoprecipitated with Flag agarose, separated by SDS PAGE, and probed for WDR48 (anti-Xpress) and EBNA3 proteins (anti-Flag). (C) Comparison of WDR48 binding results (from B) with previously published RBPJ binding results and LCL growth phenotype for each EBNA3C mutant [21].

In order to map the EBNA3 residues that mediate interaction with WDR48, additional immunoprepicitation assays were conducted using EBNA3 deletion mutants. Each EBNA3 protein was split into 3 approximately equal fragments, an N terminal region encompassing the RBPJ binding motif, a central region, and a C-terminal domain. These 3 fragments were fused to Flag and co-expressed with WDR48 in 293T cells. This revealed that EBNA3A aa524-944, EBNA3B aa394-938, and EBNA3C aa365-545 interacted with WDR48 as well or better than the full-length proteins (S4 Fig). We further mapped the EBNA3A and EBNA3C interactions using internal deletion mutants that have been assessed for their ability to support LCL growth [21,23] (Fig 6A and 6B). These data revealed that EBNA3A aa827-944 was critical for WDR48 interaction. In the case of EBNA3C, small deletions of aa447-500 or 501–544 disrupted interaction with WDR48, as did mutation of the EBNA3C SUMO interaction motif (E3C509mSIM). Importantly, EBNA3C mutants that were defective for WDR48 association correspond to mutants that are intermediate for supporting LCL growth (Fig 6C). Thus, the EBNA3 domains responsible for association with WDR48, while not as critical as the RBPJ association domains are important for EBNA3 mediated growth effects in LCLs.

### A SUMO-like domain (SLD) within WDR48 mediates binding to EBNA3B and EBNA3C, but not EBNA3A

**Fig 7:**
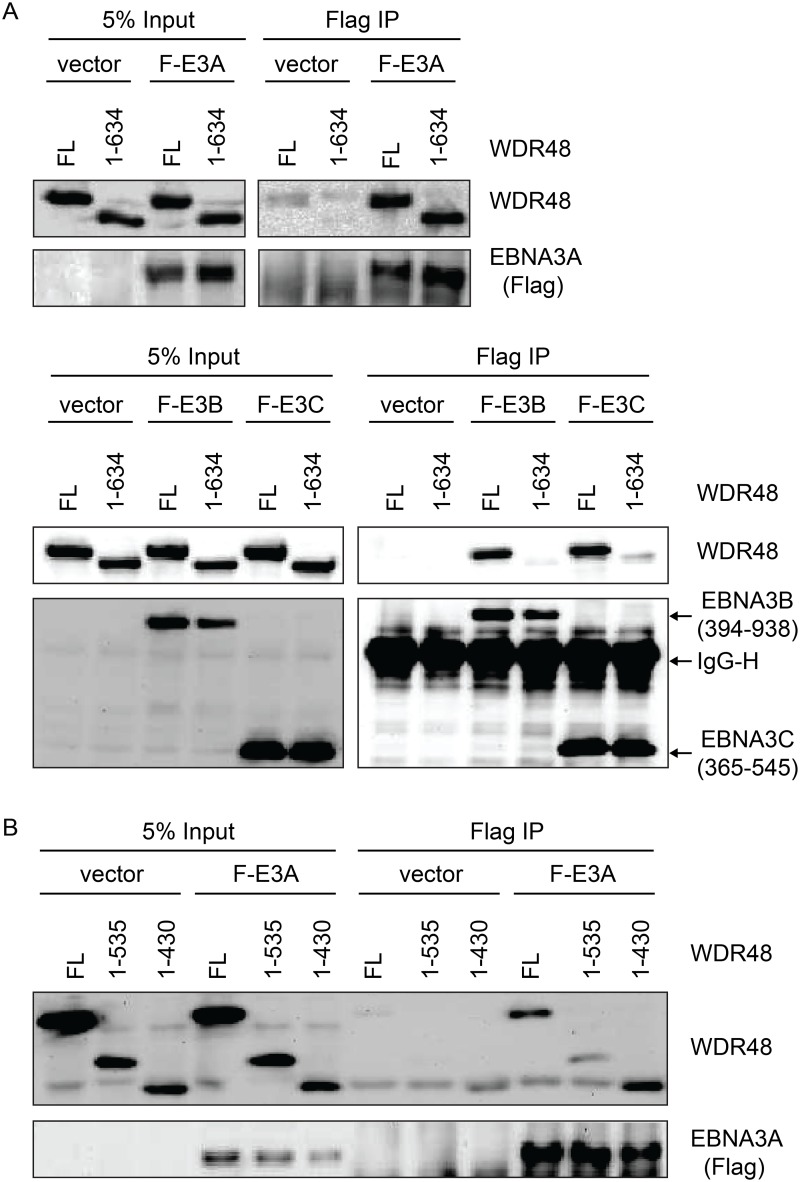
WDR48 SLD2 mediates binding to EBNA3B and EBNA3C, but is not required for EBNA3A binding. Immunoprecipitation assays were performed to assess effect of deleting the WDR48 SUMO-like domains (SLDs) on EBNA3 binding. (A) 293T cells were co-transfected with vector control, Xpress tagged full length WDR48 (FL) or a deletion mutant lacking the SLD2 domain (WDR48 1–634) and flag tagged EBNA3A 1–944 (F-E3A), EBNA3B 394–938 (F-E3B) or EBNA3C 365–545 (F-E3C). Cell lysates were immunoprecipitated with Flag agarose, separated by SDS PAGE, and probed with WDR48 or Flag antibody. (B) Immunoprecipitation assay to determine effect of deleting SLD1/2 on WDR48 binding to EBNA3A. Assays were performed as describe above with co-transfection of EBNA3A WT and either full length WDR48 (FL), WDR48 1–535, or WDR48 1–430 (which lacks both SLD1 and SLD2).

In the Fanconi anemia DNA repair pathway, SUMO interaction motifs (SIMs) of the FANCI protein associate with sumo-like domains (SLDs) within the WDR48 C-terminus [55]. Because EBNA3s proteins all contain SIMs [56], and our mapping data implicated the EBNA3C SIM in WDR48 binding, we speculated that these SLDs might be important for EBNA3A, EBNA3B, and EBNA3C interactions with WDR48. We first evaluated WDR48 aa1-634, a previously described mutant (also called WDR48ΔSLD2) that is unable to associate with FANCI [55]. Flag-EBNA3A was able to associate with this mutant comparable to wildtype WDR48 (Fig 7A). By contrast, EBNA3B aa394-938, which associates strongly with full length WDR48, did not bind to WDR48 aa1-634. The strong association of EBNA3C aa365-545 with full length WDR48 was almost completely lost with WDR48 aa1-634 (Fig 7A). To further define the EBNA3A binding site, we constructed additional WDR48 truncation mutants: WDR48 aa1-535, which removes the spacer region between SLD1 and SLD2 and WDR48 aa1-430, which is deleted for both SLDs. Both WDR48 truncation mutants interacted with EBNA3A and WDR48 aa1-430 interacted as efficiently as full length WDR48 (Fig 7B). Thus, EBNA3B and EBNA3C require the WDR48 SLD2 domain for binding, but EBNA3A can associate with the WDR48 N-terminus, independent of the SLDs.

### CtBP1 and WDR48 bind distinct residues with EBNA3A aa827-944

**Fig 8:**
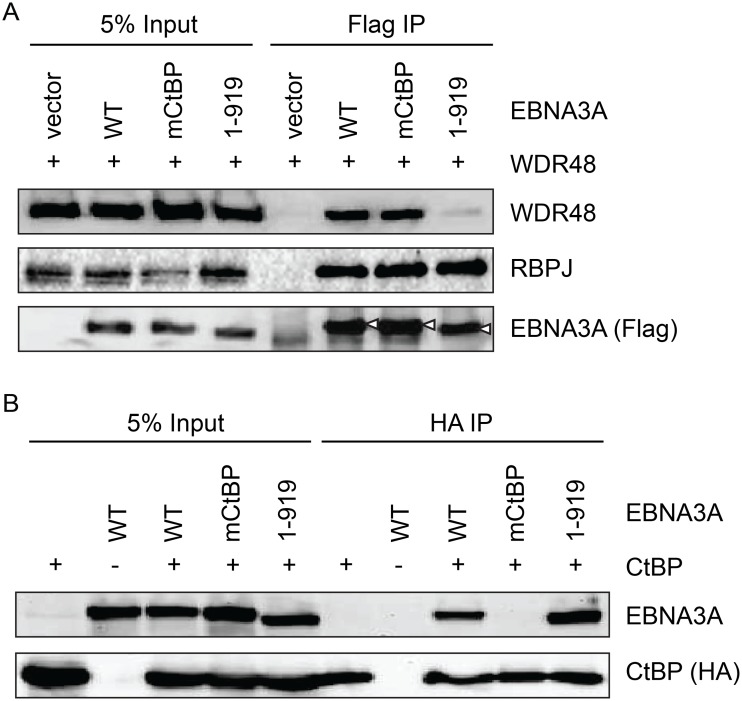
Deletion of EBNA3A residues 920–944 disrupts WDR48 binding without affecting CtBP1 association. Co-immunoprecipitation assay to assess binding of EBNA3A mutants to WDR48 (A) and CtBP1 (B). For these assays, flag tagged full length EBNA3A (1–944), an EBNA3A CtBP1 binding mutant (mCtBP), an EBNA3A mutant lacking the C-terminal 25 residues (1–919), or vector control (pSG5) was co-transfected with Xpress-WDR48 or HA-CtBP1. Lysates were immunoprecipitated with Flag agarose (A) or HA agarose (B), separated by SDS PAGE, and probed with WDR48, RBPJ, flag, EBNA3A and HA antibodies.

Because EBNA3A aa827-944, which is essential for WDR48 binding, also contains two CtBP1 binding motifs [44], we sought to determine whether CtBP1 binding was separable from WDR48 binding. Deletion of EBNA3A aa920-944 (EBNA3A 1–919) had no effect on CtBP1 or RBPJ binding, but dramatically impaired WDR48 association in co-immunoprepicitation assays (Fig 8). Immunoprecipitations also confirmed that the previously described EBNA3A CtBP1 binding mutant [44] retains the ability to bind to WDR48 with efficiency comparable to wild type EBNA3A (Fig 8B). These data demonstrated that EBNA3A aa827-944 contain distinct binding sites for CtBP1 and WDR48.

### An EBNA3A mutant defective for WDR48 association is impaired for LCL growth maintenance

**Fig 9:**
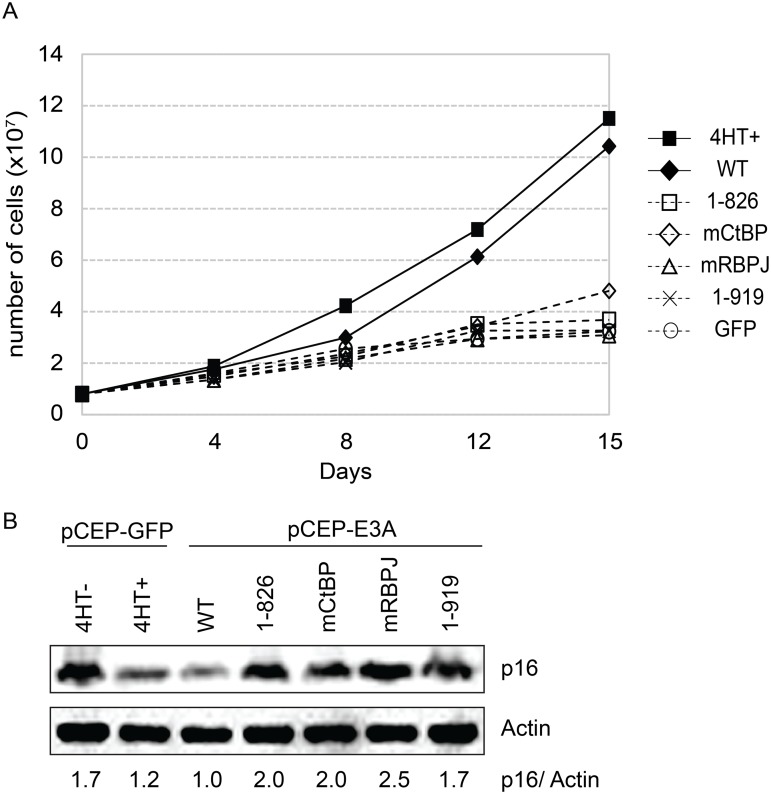
EBNA3A1-919, which associates with CtBP1 but not WDR48, is impaired for LCL growth maintenance. (A) Transcomplementation assay comparing growth of EBNA3A-HT cells transfected with EBNA3A WT (closed diamond), EBNA3A 1–826 (open square), EBNA3A mCtBP1 (open diamond), EBNA3A mRBPJ (open triangle), or EBNA3A 1–919 (X) in the absence of 4HT. EBNA3A-HT cells were also transfected with a control GFP expression plasmid, split, and maintained in either the presence (closed square) or absence (open circle) of 4HT. Cells were counted every 3 to 4 days, and diluted in fresh media to maintain a concentration of 200,000 cells/mL. Based on dilution factors, total cell number was calculated and is plotted on the Y-axis versus time. (B) Wild type EBNA3A, not mutant EBNA3A, suppresses p16 expression level in trans-complemented cells. After 15 days of EBNA3A WT or EBNA3A mutant (1–826, mCtBP1, mRBPJ, or 1–919) transfection, cells were harvested and protein expression was detected by immunoblotting with p16 antibody and actin as an internal control. As a control experiment, GFP expression plasmid was transfected into the cells and cultured with or without 4HT for 15 days. The ration of the p16 and actin bands was quantified and is indicated in the bottom panel.

In order to assess whether WDR48 binding by EBNA3A might be important for LCL growth, we tested EBNA3A aa1-919, which binds CtBP1 but not WDR48 to binding, in EBNA3A-HT LCL growth complementation. For these experiments EBNA3A-HT cells were transfected with EBNA3A, EBNA3A mutant or control GFP expression plasmid, split, maintained in growth media lacking 4HT and compared with control transfected cells grown in the presence of 4HT (Fig 9A and S5 Fig). In the presence of 4HT (closed square) or transcomplemented wt EBNA3A (closed diamond), LCL growth continued, whereas transcomplementation with EBNA3A CtBP1 binding mutant (open diamond) resulted in modestly impaired growth. By contrast, EBNA3A mutants impaired for RBPJ or WDR48 association were unable to maintain LCL growth under these conditions. Western blotting for p16 expression demonstrated that EBNA3A mutants defective for supporting LCL growth, including the WDR48 binding mutant (EBNA3A aa1-919) were impaired for suppression of p16 expression, compared to wild type EBNA3A (Fig 9B).

### Purified EBNA3 complexes exhibit DUB activity

**Fig 10:**
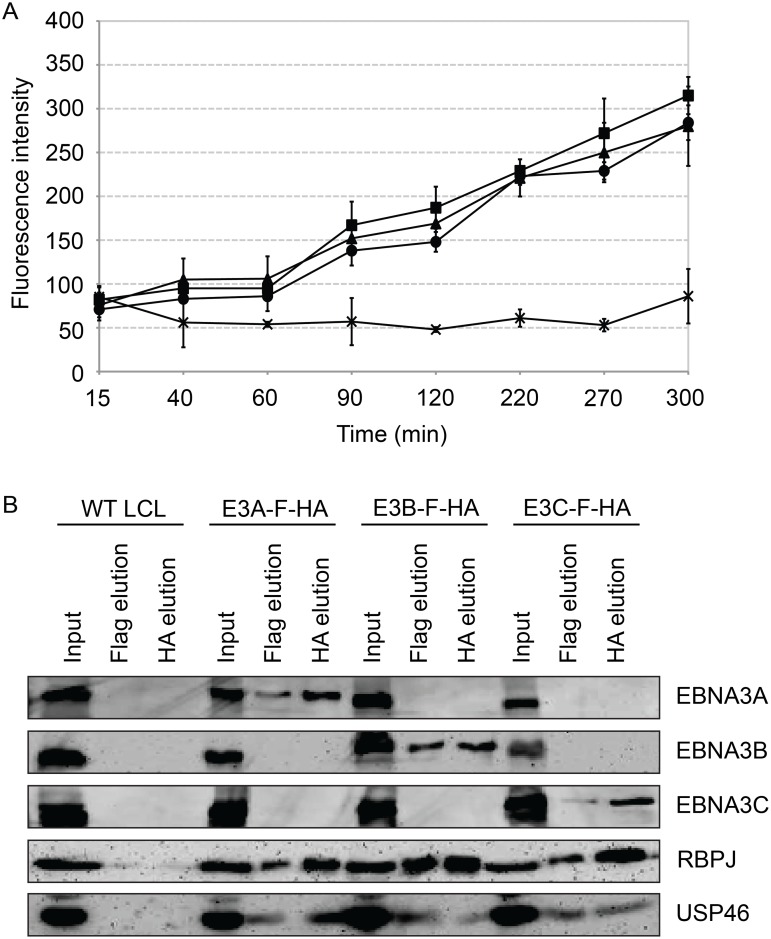
Purified EBNA3 complexes exhibit DUB activity. (A) Tandem affinity purification was performed on wild type or EBNA3-F-HA expressing LCLs. Purified complexes from wild type (X), E3A-F-HA (closed circle), E3B-F-HA (closed triangle), or E3C-F-HA (closed square) LCLs were assayed for DUB activity by fluorometric assay using Ub-AMC as a substrate. (B) Western blot of purified EBNA3 complexes used in (A) demonstrating selective precipitation of tagged EBNA3 protein complexes and co-purification of RBPJ and USP46 proteins. Lysates derived from the equivalent of approximately 1.5x10^6^ cells (input), 2.4x10^6^ cells (Flag elution), or 10x10^6^ cells (HA elution) were loaded on each lane, separated by SDS PAGE, and probed with EBNA3s, Flag, EBNA3A, and HA antibodies.

We assessed the ability of purified EBNA3 complexes to cleave ubiquitin from the 7-amino-4-methlcoumarin (AMC) fluorophore (Fig 10A) in an effort to determine whether USP46/USP12 deubiqutinase activity is activated or inhibited by association with EBNA3 proteins. EBNA3 complexes, isolated by TAP from EBNA3A-F-HA LCL (closed circle), EBNA3B-F-HA LCL (closed triangle), or EBNA3C-F-HA LCL (closed square) or control WT LCL (X), were incubated with Ub-AMC reaction buffer and fluorescence intensities were measured by fluorometer. For each EBNA3 complex, but not wt control, fluorescence intensity increased with time during the assay, consistent with DUB activity within each EBNA3 complex. Interestingly, the amount of USP46 isolated from EBNA3C-F-HA LCLs was comparable to that seen in EBNA3A or EBNA3B complexes (Fig 10B) further confirming the association of the USP46 DUB complex with EBNA3C in LCLs.

### Inability to derive USP46 null LCLs using CRISPR/Cas9 gene editing

**Fig 11:**
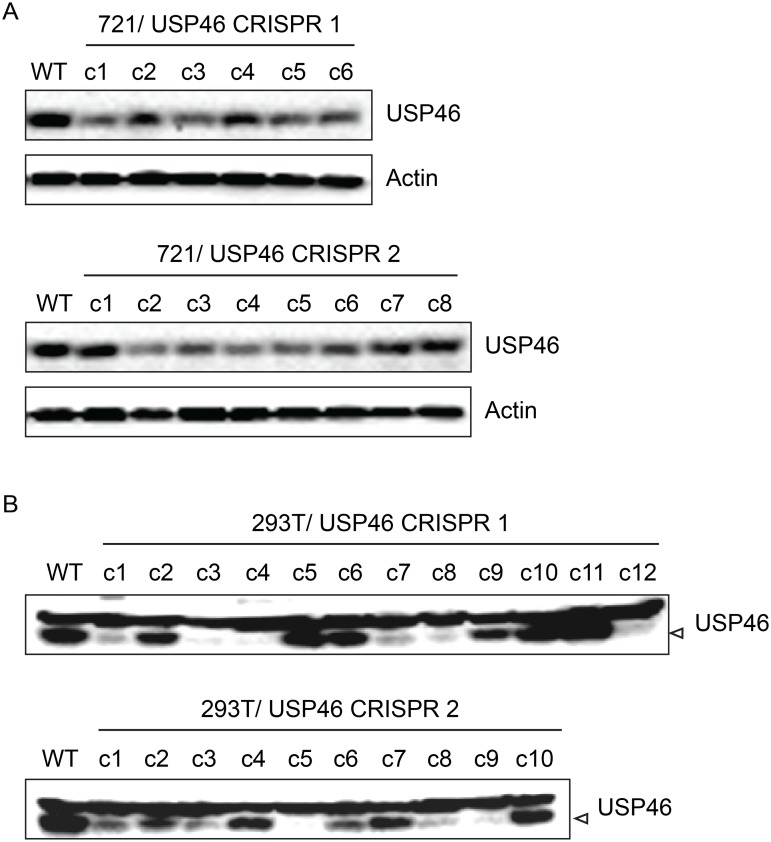
Inability to derive USP46 null LCLs using CRISPR/ Cas9 mediated gene editing. (A) Western blot for USP46 in 721 LCLs cells transfected with a plasmid expressing either of two guide RNAs targeting different USP46 exons (CRISPR 1 or CRISPR 2) and a hygromycin resistance gene. Prior to harvesting, cells were subjected to hygromycin selection for one month (resulting in 6 clones for CRISPR 1 and 8 clones CRISPR 2). Untransfected 721 cells are also shown (WT). As a loading control, lysates were probed for beta actin (bottom panel). (B) Western blots of 293T cells that were transfected same CRISPR plasmids as in panel A and also subjected to one month of hygromycin selection. Hygromycin resistance cells were harvested and blotted for USP46 (specific band is indicated by arrowhead). Untransfected 293T cells are also shown (WT).

In order to assess the requirement for USP46 expression in LCLs, we attempted to knockout USP46 expression using CRISPR/Cas9 gene editing. We cloned two guide RNA (gRNA) sequences into the pX330 vector, which allows simultaneous expression of the Cas9 nuclease and a gRNA, and transferred this dual expression cassette into pCEP4 to allow hygromycin selection. Each construct was transfected into the 721 LCL and, as a control, 293T cells. We identified multiple 293T cell populations in which no expression of USP46 could be detected (Fig 11). In some cases, low level USP46 expression was detectable, which probably reflects the oligoclonal nature of this experiment. By contrast, we observed no more than an ~50% reduction in USP46 levels in the 721 LCLs (Fig 11, top panels). To ensure that the CRISPR/Cas9 mediated gene editing had worked as intended we PCR amplified and sequenced the targeted region for each USP46 gRNA from one cell population (c3 in each case). Sequencing results demonstrated that in each population, at least one allele had undergone an in-frame deletion (S6 Fig), which would be predicted to abrogate further Cas9 cleavage, but not disrupt the USP46 open reading frame. These sequencing results confirm that the USP46 gene was successfully edited in the 721 LCLs. As a further test, we performed an independent replicate of our USP46 CRISPR/Cas9 knockout in both cell lines (S7 Fig). In 293T cells, 22 of 39 clones were successfully knocked out for USP46 expression, whereas we did not observe any USP46 knockout among 39 randomly selected clones in the 721 LCL. This difference was highly statistically significant (p < 10^–8^) by a two-tailed Fishers exact test. Our results suggest that the USP46 gene is dispensable in 293T cells, but our inability to generate USP46 null LCLs using the same approach despite evidence for Cas9 mediated cleavage, implicates USP46 in LCL growth or survival.

### Recruitment of WDR48 to the p14^ARF^ promoter is EBNA3C dependent

**Fig 12:**
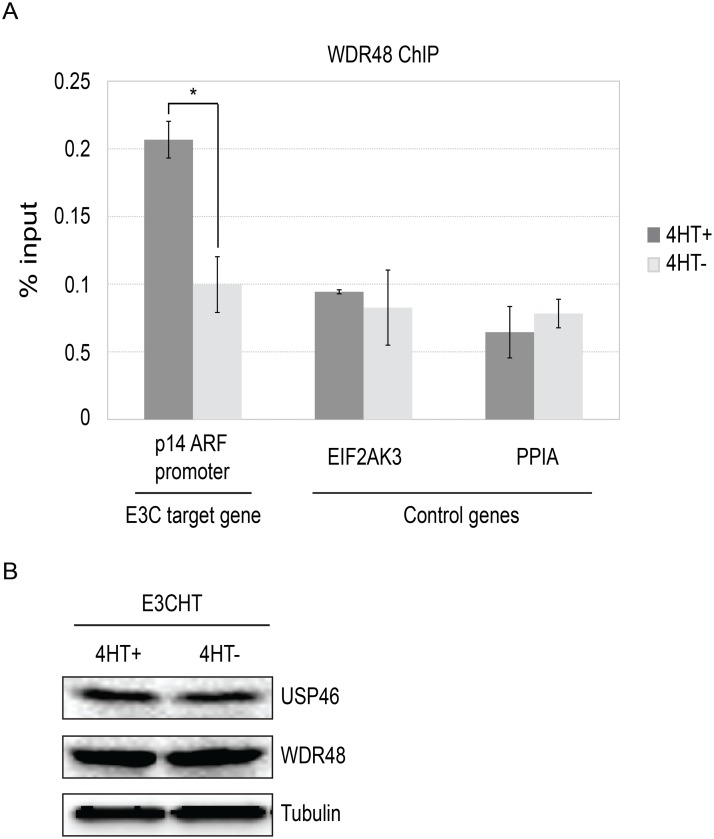
ChIP assay for WDR48 at the p14ARF promoter. Chromatin immunoprecipitation (ChIP) assays were performed using antibodies for WDR48 (A) from EBNA3C-HT LCLs that were grown in the presence of 4HT (dark gray) or after 14 days of growth in the absence of 4HT (light gray). Amount of genomic DNA was measured by real time PCR using primers specific to the EBNA3C binding site in the p14^ARF^ promoters or sites near the EIF2AK3 and PPIA genes which bind cell transcription factors but not EBNA3C. The bar graph represents the amount of DNA precipitated relative to the amount of DNA in the corresponding input sample. The experiment shown is representative of four independent experiments and error bars indicating standard error of the mean within this experiment. Asterisk denotes that the difference in ChIP signal seen at the p14^ARF^ promoter is statistically significant (p = 0.01). (B) Western blot for USP46, WDR48, and tubulin levels in whole cell lysates from EBNA3CHT LCLs grown in the presence of 4HT or after 14 days of growth in the absence of 4HT.

To more directly assess whether EBNA3C interaction with the USP46/WDR48/WDR20, could recruit the DUB complex to chromatin, we performed chromatin immunoprecipitation (ChIP) assays for WDR48 in EBNA3C-HT LCLs and assayed for enrichment using qPCR [42]. We first examined the EBNA3C binding site within the p14^**ARF**^ promoter that was recently reported to mediate recruitment of repressor complexes to this promoter. We observed an increase in ChIP signal in the presence of 4HT over that seen with 4HT withdrawal (Fig 12). As controls, we examined two additional sites located near the EIF2AK3 and PPIA genes which are bound by cell transcription factors, but not by EBNA3C [42,57]. At each of these locations, we observed no enrichment for in the permissive (4HT+) condition relative to the EBNA3C inactivation state (4HT-). Total levels of WDR48 and USP46 were unchanged upon 4HT withdrawal (Fig 12B) thus increased signal in the WDR48 ChIP at the p14 was not attributable to increased expression of the constituents of the USP46/WDR48/WDR20 complex by EBNA3C. These results are consistent with an EBNA3C dependent recruitment or stabilization of USP46/WDR48/WDR20 complex binding at the p14^**ARF**^ promoter.

## Discussion

In this manuscript, we report the first detailed characterization of EBNA3A, EBNA3B, and EBNA3C complexes from LCLs. Despite an extensive literature on putative EBNA3 interacting proteins, endogenous EBNA3 complexes have not previously been isolated and subjected to LC/MS/MS analysis. The use of epitope tags permitted tandem affinity purification of these complexes and minimized the chances that observed differences in composition were attributable to differences in the antibodies used. Our approach has additional advantages in that the proteins were expressed at endogenous levels from their native promoters. Further, because these recombinant EBV genomes were able to transform primary B lymphocytes into LCLs, the epitope tags did not disrupt EBNA3 interactions essential for the transformation process. Despite the large array of proteins reported to interact with each EBNA3, we identified only a limited number of proteins specifically associated with the EBNA3s through the TAP procedure. This limited overlap between binary protein-protein interaction screens and protein complex composition is consistent with results from large scale protein interactome mapping efforts [58]. It is a formal possibility that the purified EBNA3 complexes associated with these cell proteins during the purification procedure. We view this as unlikely since it requires the simultaneous assumption that the interaction is sufficiently strong to be maintained during the TAP procedure, but does not occur endogenously despite these proteins being present in the same subcellular fraction. One important caveat for our analysis is that the TAP procedure, while highly specific, can be insensitive for weak interacting partners. Thus, the complexes defined in our study may be most appropriately described as EBNA3 “core” complexes.

Each EBNA3 complex was found to include RBPJ, a transcription factor in the Notch signaling pathway that is critical for EBNA3A and EBNA3C function in maintaining LCL growth. It was previously known that EBNA2 and EBNA3C exist in separate RBPJ complexes [49]. Our results demonstrate that all four RBPJ-interacting EBNAs (2, 3A, 3B, and 3C) form distinct RBPJ complexes. This has several important implications for the mechanisms by which EBNA3 proteins can act to regulate transcription and their ability to modulate EBNA2 activation [38,59,60,61]. First, despite binding to a distinct domain in the RBPJ N-terminus, EBNA3 proteins are able to exclude EBNA2 which interacts with the RBPJ beta trefoil domain. Further, because EBNA3A and EBNA3C must each interact with RBPJ to maintain LCL growth via p16 promoter repression, it is likely that two different RBPJ molecules, and hence, binding sites are required. Although we did not detect stable interactions among the EBNA3 complexes, it is conceivable that interactions described by other investigators at these promoters are required for cooperative gene regulation observed among EBNA3 proteins [62]. Indeed, it is tempting to speculate that EBNA3 proteins exert their cooperative effects by exploiting paired RBPJ sites in the human genome that are important for activation of specific genes by intracellular Notch [63]. Interactions with other transcription factors are also likely to be important for observed differences in EBNA3A, EBNA3B, and EBNA3C binding observed in ChIP-seq experiments [42,57].

Although we initially embarked on these experiments with the expectation of identifying unique EBNA3A, EBNA3B, and EBNA3C interacting partners, we unexpectedly identified another shared EBNA3 target: the USP46 and USP12 deubiqutinases (DUBs) and their chaperones WDR48 and WDR20 [64,65]. Because we did not find peptides corresponding to other EBNA proteins in these complexes, each EBNA3 protein appears to target RBPJ and the USP46/USP12 DUB complexes independently. Each EBNA3 bound most strongly to WDR48, and USP46 binding was enhanced by WDR48 co-transfection, consistent with WDR48 being the primary mediator of EBNA3 binding to the USP46/USP12 DUB complexes. It is notable that EBNA3B and EBNA3C target the WDR48 SLD2 domain, whereas EBNA3A interacts with the WD repeats of WDR48. Thus, the EBNA3 proteins bind to WDR48 via their highly divergent C-termini and do not all target the same WDR48 subdomains. Whether these distinct strategies for targeting the WDR48 protein are an accident of positive selection or account for differences between EBNA3A and EBNA3C’s roles in LCL growth is not clear. These binding site differences would allow for chromatin associated EBNA3A and EBNA3C to simultaneously bind (and potentially stabilize) a single WDR48 molecule, but we found no evidence for stable binding of both EBNA3A and EBNA3C in a single complex in our LC/MS/MS data. Nevertheless, we find that USP46/WDR48/WDR20 is a component of endogenous EBNA3 complexes purified from LCLs and is bound by domains of EBNA3A and EBNA3C that are important for p16 suppression and LCL growth. Taken together these findings suggest that these DUB complexes are specifically targeted by EBNA3 proteins as part of the EBV lymphocyte transformation strategy.

The ubiquitin specific proteases USP12 and USP46 are close paralogs, that are 89% identical over 357 residues and are both regulated by the WDR48 and WDR20 chaperones [64,66]. Although the physiologic role of these enzymes is incompletely understood, they appear to exhibit partially overlapping substrate specificity [64]. The more distantly related USP1 is also regulated by WDR48, but WDR20 is unique to USP12/USP46 complexes. We did not detect any USP1 peptides in our complexes, suggesting that the even though the EBNA3 proteins interact strongly with WDR48, they are selective for USP12 and USP46 complexes, possibly due to stabilizing interactions with WDR20 or the enzymes themselves. Although the PHLLP1 and PHLLP2 phosphatases have been reported to be components of the USP46 and USP12 complexes, they were not detectably associated with EBNA3 complexes, likely because these phosphatases are predominantly membrane-associated [67,68,69,70]. Because regulation of the steady state levels of the PHLLP phosphatases by USP46/USP12 is a critical regulatory step in the Akt signaling pathway, EBNA3 proteins might influence PHLLP protein levels, and hence, alter Akt signaling by binding this DUB complex. However, PHLLP1 was not detectable in our LCLs and we found no evidence that PHLLP2 levels or Akt phosphorylation were effected by EBNA3C inactivation (S8 Fig). Further, USP46 complexes were present in both the cytoplasm and the nucleoplasm of LCLs and this distribution was also observed in EBV negative B cells. Membrane associated USP46 and USP12 complexes have been implicated in regulating membrane trafficking of receptors, including Notch1 and GLR1 [71,72]. Although it is possible that EBNA3 proteins could affect this regulation, we do not favor this hypothesis as the levels of membrane associated USP46 in LCLs are not reduced, but slightly higher than that observed in EBV negative BL41 cells.

Our inability to derive USP46 null LCLs using CRIPSR/Cas9 gene editing is consistent with USP46 playing an essential role in LCL growth or survival that it does not play in 293T cells. Based on our observation that WDR48 plays a dominant role in mediating EBNA3 association with the USP46 DUB complex and binds to EBNA3A and EBNA3C domains that are important for regulation of p16, we suspect that the DUB complex interaction is important for transcriptional effects of the EBNA3 proteins. We considered the possibility that this interaction contributes to the long half-life of EBNA3 proteins; however the steady state levels of EBNA3A and EBNA3C WDR48 binding mutants were not detectably different than wild type (Fig 6) and there was no detectable difference in protein turnover in the presence of cyclohexamide (S9 Fig). Instead, our results suggest that EBNA3 proteins act as adaptor molecules to target USP46 complexes to promoters via interactions between RBPJ and other transcription factors. This is supported by our observation that WDR48 is recruited the p14^ARF^ promoter in an EBNA3C dependent manner. Given the central role of ubiquitylation in transcriptional activation, we favor the hypothesis that the EBNA3 proteins recruit the DUB complex to remove ubiquitin molecules from other nuclear proteins. However, we are unaware of any unbiased methods for determining the substrates of DUB complexes. Using a candidate substrate approach, we investigated the possibility that ubiquitylation of histone H2A (H2A-Ub) and H2B (H2B-Ub) were targeted by these complexes as has been previously described in Xenopus [73]. However, we found global levels of H2A-Ub and H2B-Ub to be unaffected by the presence EBNA3 proteins (S10 Fig). We did observe decreased H2A-Ub at the p16 promoter upon EBNA3C-HT inactivation (S10 Fig) and no change in H2B-Ub levels. This is consistent with decreased polycomb repression of p16 upon EBNA3C-HT inactivation, but not consistent with recruitment of USP46 to chromatin playing a direct role in deubiquitylating histones at the p16 promoter.

In summary, we find that the USP46/USP12 DUB complexes are a highly associated with EBNA3 proteins in LCLs, interact with domains of EBNA3A and EBNA3C essential for LCL growth, and that DUB activity is preserved in these complexes. The substrates upon which these DUBs act upon in LCLs remain to be determined, despite our efforts to identify effects of the EBNA3 proteins on several candidates. Although we have focused on transcriptional effects of the EBNA3 proteins, it is likely that their ability to associate with the USP46/USP12 DUB complexes explains other observed EBNA3 activities as well, most notably their effects on the stability of cell proteins, including Mdm2, cyclin D, Gemin3, IRF4 or aurora kinase B [27,30,31,74,75]. We believe that the identification of the USP46/USP12 DUBs as components of the EBNA3 complexes in LCLs represents a significant advance in our understanding of the multitude of roles played by EBNA3 proteins in B lymphocyte transformation.

## Materials and Methods

### Ethics statement

Lymphoblastoid cell lines (LCLs) described in this manuscript were derived by EBV transformation of peripheral blood B lymphocytes from de-identified donors, with written informed consent, which is approved by Partners IRB based on Helsinki recommendations.

### Cell lines

293T (obtained from Elliott Kieff, Harvard Medical School), a human cell line transformed by adenovirus 5 and SV40 large T antigen [76], was cultured in Dulbecco’s modified Eagle’s (Gibco) medium. The “wild-type” LCL, created with an unmodified EBV BACmid, was a generous gift from Fred Wang [47] and the 721 LCL was obtained from Bill Sugden [77]. LCLs and P3HR1 ZHT cells [78], a type II EBV cell line, were cultured in RPMI 1640 (Gibco). Media was supplemented with L-glutamine (Gibco), penicillin-streptomycin (Gibco) and 10–15% FetalPlex (Gemini Bio-Products).

### Plasmids

pBS-XS-EA contains the XbaI-SalI fragment from the EBV B95-8 genome containing the EBNA3C region, in which the C-terminus of the EBNA3C ORF is mutated to create EcoRI and AvrII sites (GATTCGATTAAGGGGATCCTAGG). pBS-EBNA3C-flag-HA-CAT was created from pBS-XS-EA by inserting an oligo encoding the flag and HA epitopes (AATTGGATGAATTCGCGGCCGCTGGAGGAGACTACAAGGACGACGATGACAAGTCGGCCGCTGGAGGATACCCCTACGACGTGCCCGACTACGCCTAGGACGCGT annealed to CTAGACGCGTGGATCCGCATCAGCCCGTGCAGCATCCCCATAGGAGGTCCGCGGCTGAACAGTAGCAGCAGGAACATCAGAGGAGGTCGCCGGCGCTTAAGTAGG) and a PCR product containing an FRT flanked CAT gene amplified from pKD3 using the primers (CACTGAATTCCTAGGTAGGTGTAGGCTGGAGCTGCTTCGAAG and TTGAATGAACGCGTCATATGAATATCCTCCTTAG). pSG5-EBNA3A-flag-HA-CAT and pSG5-EBNA3B-flag-HA-CAT were created by cloning the NotI/MluI fragment from pBS-EBNA3C-flag-HA-CAT into pSG5-EBNA3A and pSG5-EBNA3B which had been modified to create NotI/MluI sites allowing the flag-HA tag to be fused in-frame with the EBNA3A or EBNA3B ORFs, respectively. Plasmids for expression of EBNA3A and EBNA3C mutants have been previously described [21,23]. pSG5-flag-EBNA3A 1–919 was constructed by PCR amplify of pSG5-Flag-EBNA3A using primer pairs EBNA3A-C919 (ACAACAGCTGGCGGCCGCTACCTTCTAGTTTCAGGGCCTGTGACATTTTGGCCAC) and EBNA3A-N543 (CTCAGGGAATGGCATACCCATTAC), digested with NotI/BssHII and recloned into pSG5-Flag-EBNA3A or pCEP-Flag-EBNA3A. pSG5-Flag-E3A mCtBP1 was constructed same as previously described [44]. Flag-tagged EBNA3B 1–938, 1–544, and 394–938 were constructed by PCR amplification from pSG5-EBNA3B [23] using appropriate pairs of the following primers: E3B-N1 (TTGTACAAAACTGCAGGCATGAAGAAAGCGTGGCTCAG), E3B-C938 (AACTTTGTACGCGGCCGCTTACTCATCGTTCGATGTTTCAGAAG), E3B-C544 (TCACTCTCTAGCGGCCGCTAACCGGTGAAGACACAAGGGCCTC), and E3B-N394 (CTGCCGTACACTGCAGCAGTATACGGCAGGCCCGCGGTG), and cloned into the PstI/NotI sites of pSG5-flag [79]. The expression construct for Xpress-tagged-WDR48 was a kind gift of Jae Jung [51]. WDR48 1–634 (ΔSLD2) was constructed using WDR48-N399 (GCAAAGTGGATTTTGAAGATG) and WDR48-C634 (AGTTCAATTGCGGCCGCCTACAACACAGCAATATCTTCTTC). Resulting PCR product was digested with HpaI/NotI and recloned into pcDNA4-WDR48. WDR48 1–535 (ΔSLD2) was constructed using WDR48 C535stop (TGTTTCATTAAGCGGCCGCTACGTTAACTAACCCCCGGAATCTCGGCAGAGCAGC) and WDR48-N295 (GCACCAGTTCTCAAGATGGAGC). Resulting PCR product was digested with EcoRI/NotI and recloned into pcDNA4-WDR48. Then WDR48 1–535 (ΔSLD2) was digested with HpaI and religated to make WDR48 1–430 (ΔSLD1/2). Xpress tagged USP46 and WDR20 were constructed by amplifying the ORFs from ORFome 5.1 Entry clones (generous gifts of Marc Vidal) using the following primers: TGTACAAAAGGTACCTATGACTGTCCGAAACATCGCCTC + CTTTGTACTCGAGCGGCCGCTATTCTCTTGACTGATAGAATAAAATATATC and TGTACAAAAAGGTACCTATGGCGACGGAGGGAGGAGGGAAG + AACTTTGTACTCGAGCGGCCGCTAAGGATTAAAACTTACCACTTTACCAG, respectively. Resulting PCR products were KpnI/NotI digested and cloned into pCDNA4-HisMax-A (Invitrogen). All constructs were verified by sequencing.

### Recombinant EBV genomes

In frame C-terminal flag-HA tags were fused to the EBNA3A, EBNA3B, or EBNA3C ORFs as follows: DNA fragments containing these fusions were either excised as an Xba/SalI fragment (from pBS-E3C-flag-HA-CAT) or PCR amplified (from pSG5-E3A-flag-HA-CAT and pSG5-E3B-flag-HA-CAT), using the E3A-F (TGACGTGGTCCAACATCAGC) and E3A-R (GCGTATTATCAGTGGGTGGAATGGAGGGGGACACACTTCTACACCTTTGCCATATGAATATCCTCCTTAG) or the E3B-F (ACTCCCATGCAGCTGGCACTAAGGGC) and E3B-R (CCCCGCAGTCTGTTGCCCCAGGGTTCATCCCAGTTCTTGTTACATGGGCGCATATGAATATCCTCCTTAG) primers. Fragments were recombined into an EBV-BACmid derived from the B95-8 genome using an inducible lambda red recombinase as previously described [47]. Following transient expression of FLP recombinase, single colonies were plated and screened for excision of the CAT gene.

### LCL transformation using recombinant EBV genomes

Recombinant EBV-BACmids were transfected into P3HR1 ZHT cells, selected with puromycin and induced for replication by addition of 4HT. Viral supernatant were collected and used to transform peripheral blood B cells in to lymphoblastoid cells as previously described [23]. LCLs were screened by PCR for recombinant genomes containing the flag-HA fused in frame to the appropriate EBNA3 open reading frame and the absence of co-infecting P3HR1 genomes.

### Antibodies

The following antibodies were used for Western blotting, immunoprecipitations and chromatin immunoprepicitation: mouse monoclonal antibodies against HA.11 (16B12, Covance), Flag (M2 and M5, Sigma), Xpress (R910-25, Invitrogen), alpha-tubulin (B-5-1-2, Sigma), Glyceraldehyde-3-Phosphate Dehydrogenase (GAPDH; 6C5, Millipore), Beta Actin (Sigma, A5441), BRG1 (PA5-17008, Thermo Scientific), LaminB (sc-6216, Santa Cruz), RBPJ (Hyb-T6709, Cosmo Bio Co), CtBP1 (Q13363, Millipore), WDR48 (HPA038421, SIGMA or rabbit polyconal sera, a kind gift of Alan D’Andrea), WDR20 (A301-657A, Bethyl Laboratories), USP46 (HPA007288, Sigma), p16 (clone JC8, sc-56330, Santa Cruz Biotechnology), NF-kB p65 (8242, Cell Signalling), EBNA1 (13-156-100, Advanced Biotechnologies), EBNA2 (PE2, [80]), EBNA3A (F115P, Exalpha Biologicals), EBNA3B (F120P, Exalpha Biologicals), EBNA3C (A10, [81]), LMP1 (S12, [82]), EBNALP (4D3, a kind gift of Yasushi Kawaguchi [83]). histone H2A (#07–146, Millipore), Ub-Histone H2A (#05–678, Millipore), histone H2B (07–371, Millipore), Ub-Histone H2B (#07–371, Millipore), PHLPP1 (A300-660A, Bethyl), PHLPP2 (A300-661A, Bethyl), Akt (#4691, Cell signaling), p-Akt (#4060, Cell signaling), and GFP (sc-5384, Santa Cruz).

### Tandem-affinity purification (TAP)

Approximately 6x10^8^ cells from an LCL transformed with either a wild-type EBV BAC or a recombinant EBV BAC expressing either EBNA3A-F-HA, EBNA3B-F-HA, or EBNA3C-F-HA were lysed in 10ml of TAP lysis buffer (1% (v/v) Igepal CA-630, 50mM TrisHCl [pH7.5], 140mM KCl, 10mM NaF, 1.5mM EDTA, and 5% glycerol) containing 10mM β-ME and EDTA-free Complete protease inhibitor (Roche, Mannheim, Germany). Lysed cells were incubated at 4°C for 30 minutes with constant rotation before being cleared by two rounds of centrifugation at 8500rpm for 10 minutes and one spin at 10,000rpm for 20 minutes. Supernatants were diluted as required to match total protein concentration as measured by Bradford assay (BioRad). A 50μl aliquot was saved for Western blot analysis and the remaining supernatants were incubated with 60μl of anti-Flag M2 agarose (Sigma) for 4 hours at 4°C with rotation. The beads were washed extensively with TAP lysis buffer before being eluted with 60μl of 0.4mg/ml Flag peptide (Sigma) in TAP buffer twice at 4°C for 30 minutes with shaking and once at 37°C for 30 minutes with shaking. Elutions were passed through Bio-Spin columns (Bio-Rad) to remove entrained agarose beads and pooled. Agarose-conjugated HA beads, 25μl per sample, (F7, Santa Cruz Biotechnology) were added to the pooled elutions and incubated overnight at 4°C with constant rotation. The beads were washed three times with TAP lysis buffer and eluted with 30μl of 0.4mg/ml HA peptide (Covance) twice at 37°C for 30 minutes with shaking; elutions were spun through Bio-Spin columns and pooled for LC/MS/MS analysis.

### TAP mass-spectrometry analysis

Eluted samples (50μl) were mixed with 10μl of 4X LDS Loading Buffer (Invitrogen) separated on a 10% Bis Tris NuPAGE MOPS gel (Invitrogen). Gels were fixed in destain solution (50% methanol and 7.5% acetic acid), rehydrated, stained with Simply Blue Safestain (Invitrogen), cut horizontally into one slice per sample, and destained until transparent. Gel slices were reduced with DTT, alklyated with iodoacetamide, and then rinsed with three alternating washes of 50 mM ammonium bicarbonate and acetonitrile. Each slice was then digested with trypsin by resuspending in 50mM ammonium bicarbonate/10% acetonitrile/5.5g/mL trypsin and incubating at 37°C for 24 hours. Peptides were extracted with one rinse of 50mM ammonium bicarbonate/10% acetonitrile followed by one rinse of 50% acetonitrile/0.1% formic acid, lyophilized, then rehydration in 20μL 96% water, 4% methanol, and 0.2% formic acid.

Digested samples were loaded into 96-well plates for mass spectrometry analysis on a LTQ-Velos Orbitrap XL (Thermo Fisher Scientific) instrument. For each run, 10μL of each re-constituted sample was injected onto an Easy nLC system configured with a 10cmx100um trap column and a 25cm x 100um ID resolving column (Thermo Scientific). Buffer A was 96% water, 4% methanol, 0.2% formic acid and Buffer B was 10% water, 90% acetonitrile, and 0.2% formic acid. Samples were loaded at 5μL a minute for 9 minutes, and a gradient from 0–60% B at 375nl/minute was run over 70 minutes, for a total run time of 115minutes (including regeneration, and sample loading). Velos-Orbitrap (Thermo Scientific) was run in a standard data dependent Top 10 configuration at 60K resolution for a full scan, with monoisotopic precursor selection enabled, and +1, and unassigned charge state rejected. MS2 fragmentation and analysis was performed in the ion trap using CID fragmentation.

Peptides were identified using SEQUEST (Thermo Fisher Scientific) through Protein Discoverer, version 1.2. MS/MS data were searched using 10ppm mass accuracy on precursor m/z and a 0.5Da window on fragment ions. Fully enzymatic tryptic searches with up to three missed cleavage sites were allowed. Oxidized methionines were searched as a variable modification and alkylated cysteines were searched as a fixed modification. Sequential database searches were performed using the NCBI RefSeqHuman FASTA database. Peptides for each charge state were filtered to a false discovery rate (FDR) of 1%.

### Subcellular fractionation

Subcellular fractionation was performed using the subcellular protein fractionation kit (Thermo Scientific Pierce) according to the manufacturer’s instructions. For each fraction, an amount corresponding to that derived from 400,000 cells was resolved by SDS-PAGE and probed for EBNA3 proteins, RBPJ, and components of the USP46 complex (USP46, WDR20, and WDR48). Fraction purity was assessed by probing for tubulin, BRG1, Histone H2B, and LaminB.

### TAP Ub-AMC assay

In vitro deubiquitination assays using TAP purified EBNA3s complex and Ub-AMC (U-550, Boston Biochem) as a substrate were performed in 100uL of reaction buffer (20 mM HEPES-KOH at pH 7.8, 20 mM NaCl, 0.1 mg/mL BSA, 0.5 mM EDTA, 20mM beta-mercaptoethanol). Fluorescence signal was monitored in VICTOR X5 multilabel plate reader (Perkin Elmer).

### Immunoprecipitation

Transfected 293T cells which were harvested from 10cm tissue culture dishes or ten million of LCLs were lysed into IP lysis buffer (1% (v/v) Igepal CA-630, 40mM TrisHCl [pH7.5], 150mM NaCl, and 10mM MgCl_2_) supplemented with fresh 0.015mg/mL aprotinin (Sigma), 0.5mM PMSF, and 1ug/ml Leupeptin. Lysates were incubated at 4°C for 30 minutes with rotation and cleared by centrifugation at 10,000x g for 15 minutes. Supernatants were pre-cleared by rotating with Sepharose (Sigma) for 1 hour at 4°C and then incubated with anti-Flag M2 agarose, anti-HA magnetic beads (cloneTANA2, MBL), or protein A/G for 2 hours at 4°C with rotation. The beads were washed extensively with IP lysis buffer and either eluted with 0.4 mg/ml Flag peptide or 0.4mg/ml HA peptide in IP lysis buffer at 37°C for 30 minutes with shaking or resuspend into SDS sampling buffer. The proteins were analyzed by Western blotting.

### Western blot analysis

Total-cell lysates or immunoprecipitated proteins were separated by sodium dodecyl sulfate (SDS)-polyacrylamide gel electrophoresis, blotted onto nitrocellulose membrane, and probed with appropriate antibodies. After extensive washing, horseradish peroxidase conjugated secondary antibodies (Jackson Immuno Research) were applied. After incubation for 1–2 hours the membrane was washed again, and developed with chemiluminescence reagent (Perkin Elmer). Western blots were exposed on film and visualized on a KODAK Image Station 4000R (Kodak Molecular Imaging Systems).

### Complementation assay

Five million of EBNA3AHT-infected LCLs [23] were transfected with 2ug of *oriP* plasmid DNA expressing EBNA3A, EBNA3A mutant or control plasmid. LCLs were harvested during log-phase growth, washed with complete medium, resuspended in 100ul of buffer V with DNA in a cuvette, transfected using program X-001 of Amaxa Nucleofector (Lonza), and cultured for 3 days in LCL-conditioned medium with 4-hydroxy-tamoxifen (4HT). Cells were then washed with PBS twice, and cultured in complete medium with or without 4HT. Every 4 to 7 days, cell numbers were counted, cultures were split, and the total numbers of viable cells relative to those of the initial culture were calculated.

### CRISPR/ Cas9 plasmids and knockout USP46 gene

Cas9 mediated editing of the USP46 gene was accomplished by cloning either of two targeting 20mers for the gRNA (CRISPR-1 in exon3: AAACTTGCTGACGTGCCTGG and CRISPR 2 in exon4: TATTGCGGACATCCTTCAGG) [84,85] into the pX330 plasmid [86]. The Cas9 expression cassette and gRNA were excised from pX330 by PciI/ NotI digestion and cloned into pCEP4 with a modified polylinker sequence, which allowed for hygromycin selection via an self-maintaining episomal plasmid.

Five million of EBV transformed LCLs were harvested during log-phase growth, resuspended in 100ul of buffer Ingenio with pCEP-CRISPR-USP46 plasmid, transfected using program U-001 of Amaxa Nucleofector, and cultured for 2 days in RPMI1640 complete medium. 20,000 cells were plated on 96 well culture plate using RPMI1640 complete medium with 300ug/ml hygromycin for one month. For 293T cells, one million cells were transfected with pCEP-CRISPR-USP46 plasmid using Effectene, recovered for 48 hours, and then subjected to hygromycin selection. Hygromycin resistance cells were harvested and screened with DNA PCR using primer pairs (CRISPR-1 F: GGTGAGCTGGACTCCAATACAGGG and R: GCCAGCTCTTCCTTTTGAGGAGAT or CRISPR-2 F: GGAGGCAGAGGTTGCAGTGAACTG and R: GCAATCACATGCAACATAGCGTAC) and Western blotting analysis. These primers were also used for Sanger sequencing of PCR products. USP46 Western blot signals were quantified and normalized to tubulin signal. For statistical analysis any cell line exhibiting >25% normalized USP46 signal was considered positive.

### Chromatin immunoprecipitation (ChIP)

ChIP assays were performed as described previously [87]. Briefly, 2x10^6^ cells per ChIP were fixed in 1% (wt/vol) formaldehyde and sonicated using cup horns sonication system (Qsonica). After extract clearing by centrifugation, supernatants were diluted and incubated with protein G agarose with salmon sperm DNA (Millipore) for 1 h with rotation at 4°C. Protein G agarose was pelleted and supernatants were used in ChIP experiments. One or two micrograms of antibody were added per 2x10^6^ cells, followed by incubation overnight at 4°C with rotation. Purified DNA was quantified using gene specific primers and iTaq universal SYBR green supermix (Bio-Rad) using a CFX96 touch real-time PCR detection system (Bio-Rad). Primers used for these experiments were as follows: p16 TSS [32], p14^**ARF**^ [42], EIF2AK3 (F: CTTCCGGACGCAATTACCAATGAG and R: GTAGGAAAGGTATTCCGGGAACTG) or PPIA [57].

## Supporting Information

S1 Fig Characterization of LCLs transformed by recombinant EBV genomes expressing epitope-tagged EBNA3A, EBNA3B, or EBNA3C. Western blot demonstrating EBV latent protein expression in wild-type (WT), EBNA3A-Flag-HA (E3A-F-HA), EBNA3B-Flag-HA (E3B-F-HA), or EBNA3C-Flag-HA (E3C-F-HA) LCL is shown. Total cell lysates were separated by SDS PAGE and probed with antibodies for EBV latent proteins (EBNA-1, -2, -3A, -3B, -3C, -LP, or LMP1), RBPJ, or GAPDH.
(TIF)

S2 Fig Efficiency of TAP lysis procedure for extracting EBNA3s and DUB complex components. LCLs were lysed in TAP lysis buffer as detailed in the methods section and the residual pellet resuspended as a separate fraction in 1x SDS sample buffer. Total cell lysates, TAP buffer soluble (Lysate) or insoluble (Pellet) fractions were separated by SDS PAGE and probed for EBNA3s, RBPJ, WDR48, WDR20, or USP46 using appropriate antibodies. Fractions were also assessed for control proteins: tubulin (cytoplasm), BRG1 (chromatin associated), Histone H2B (chromatin), and LaminB (cytoskeleton).
(TIF)

S3 Fig USP46 distribution in EBV positive and negative cells. Subcelluar fractionation was performed as for Fig 3 in LCLs, EBV negative BL41 cells and BL41 cells exogenously infected with EBV. Subcellular fractions were blotted with the indicated antibodies. Results of two independent experiments are shown.
(TIF)

S4 Fig Coarse mapping of WDR48 binding domains of EBNA3A, EBNA3B, and EBNA3C. Immunoprecipitation assay to identify WDR48 binding regions with EBNA3A (panel A), EBNA3B (panel B), or EBNA3C (panel C). 293T cells were co-transfected with Xpress tagged WDR48 and flag tagged EBNA3A, EBNA3B, EBNA3C, or the indicated deletion mutants. Lysates were immunoprecipitated with Flag agarose, separated by SDS PAGE, and probed with for Xpress (WDR48) and Flag (EBNA3) antibody as indicated.
(TIF)

S5 Fig EBNA3A1-919, which associates with CtBP1 but not WDR48, is impaired for LCL growth maintenance. Complementation assays were performed as described in Fig 9 in EBNA3A-HT LCLs. Results of two independent experiments are shown. Growth curves for cells transfected with the following EBNA3A expression plasmids and maintained in the absence of 4HT are shown: EBNA3A WT (closed diamond), EBNA3A 1–826 (open square), EBNA3A mCtBP1 (open diamond), EBNA3A mRBPJ (open triangle), or EBNA3A 1–919 (X) in the absence of 4HT. EBNA3A-HT cells were also transfected with a control GFP expression plasmid, split, and maintained in either the presence (closed square) or absence (open circle) of 4HT.
(TIF)

S6 Fig Sequence analysis of the USP46 knockout LCLs. To determine CRISPR-Cas9 editing of the USP46 gene was sucessful in the 721 LCL, we sequenced PCR products from primers flanking the targeted 20mer from one clone for each gRNA. Shown the resultant sequences traces corresponding to 721/ UPS46 CRIPSR1 cln3 and 721/ UPS46 CRIPSR2 cln3 and their interpretations.
(TIF)

S7 Fig Inability to derive USP46 null LCLs using CRISPR/ Cas9 mediated gene editing. (A) Western blot for USP46 in 721 LCLs cells transfected with a plasmid expressing either of two guide RNAs targeting different USP46 exons as was done in Fig 11. Untransfected 721 cells are also shown (WT). As a loading control, lysates were probed for tubulin (bottom panels). (B) Western blots of untrasnfected 293T cells (WT) or 293T cells that were transfected same CRISPR plasmids and also subjected to one month of hygromycin selection. Cell lines in which USP46 expression was successfully knocked-out are circled.
(TIF)

S8 Fig Effect of EBNA3C inactivation on the PHLPP/Akt pathways. EBNA3C-HT LCLs were grown in the presence of 4HT or harvested at the indicated times after 4HT withdrawal. Levels of PHLPP1, PHLPP2, Akt and phoso-Akt were determined by immunoblotting as indicated.
(TIF)

S9 Fig The stability of mutants of EBNA3A and EBNA3C that do not bind USP46 is unaltered. Cells were treated with cycloheximide to determine whether USP46 binding alters EBNA3A or EBNA3C protein stability. EBNA3A wild type, EBNA3A mCtBP1, EBNA3A 1–919 (ΔWDR48), EBNA3C wild type, or EBNA3C 509mSIM was contranfected with destabilized GFP plasmid into 293T cells. After 24 hours cells were treated with 10ug/ml of CHX (0h) and harvested indicated time. Lysates were separated by SDS PAGE and probed with EBNA3A or EBNA3C, GFP, and Tubulin antibodies.
(TIF)

S10 Fig Effect of EBNA3C inactivation on histone ubiquitylation at p16 promoter. (A) Western blots to detect levels of global H2A and H2B ubiquityaltion in 293T cells transfected with USP46 with or without cotransfected EBNA3A or EBNA3C. (B) Immunoblots demonstrating the effect of inactivation of EBNA3C by 4HT withdrawal in E3C-HT LCLs on global levels of H2A and H2B ubiquitylation. (C) ChIP-assay examining the effect of EBNA3C inactivation in E3C-HT cells on H2A-Ub and U2B-Ub levels at the p16 promoter. This experiment is typical of three independent experiments.
(TIF)

S1 Table EBNA3A LC/MS/MS data. Result file from SEQUEST analysis of tandem affinity purified EBNA3A complexes exported into excel file format.
(XLS)

S2 Table EBNA3B LC/MS/MS data. Result file from SEQUEST analysis of tandem affinity purified EBNA3B complexes exported into excel file format.
(XLS)

S3 Table EBNA3C LC/MS/MS data. Result file from SEQUEST analysis of tandem affinity purified EBNA3C complexes exported into excel file format.
(XLS)

S4 Table Control wtLCL LC/MS/MS data. Result file from SEQUEST analysis of tandem affinity purification from control (wildtype) LCL exported into excel file format.
(XLS)
