# Supplementary figures and images for: The EBNA3 Family of Epstein-Barr Virus Nuclear Proteins Associates with the USP46/USP12 Deubiquitination Complexes to Regulate Lymphoblastoid Cell Line Growth
Source: PLoS Pathog. 2015 Apr 9;11(4):e1004822. doi: 10.1371/journal.ppat.1004822 (PMC4391933; doi:10.1371/journal.ppat.1004822)

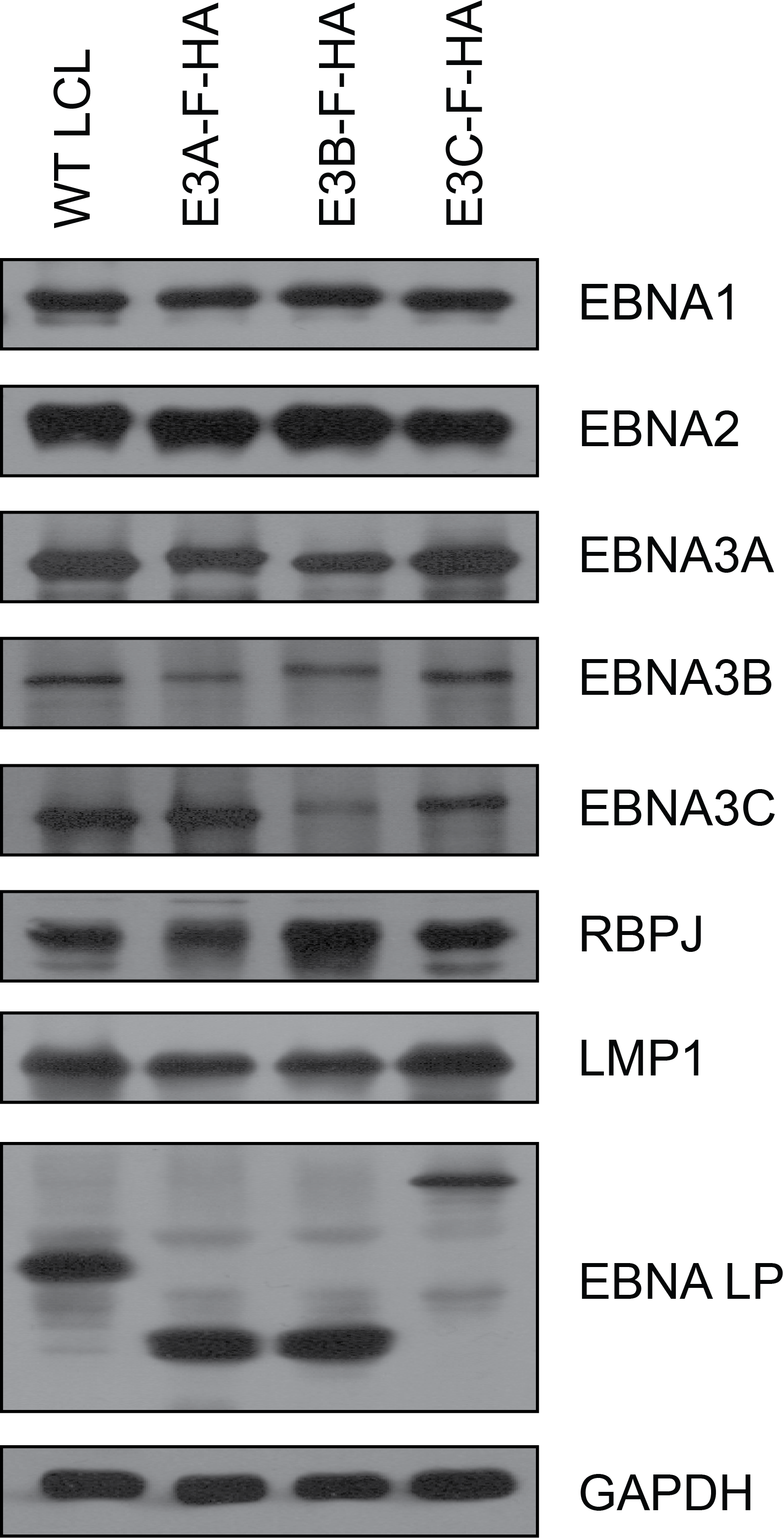

Supplement: S1 Fig — Western blot demonstrating EBV latent protein expression in wild-type (WT), EBNA3A-Flag-HA (E3A-F-HA), EBNA3B-Flag-HA (E3B-F-HA), or EBNA3C-Flag-HA (E3C-F-HA) LCL is shown. Total cell lysates were separated by SDS PAGE and probed with antibodies for EBV latent proteins (EBNA-1, -2, -3A, -3B, -3C, -LP, or LMP1), RBPJ, or GAPDH. (TIF) [file ppat.1004822.s001.tif]

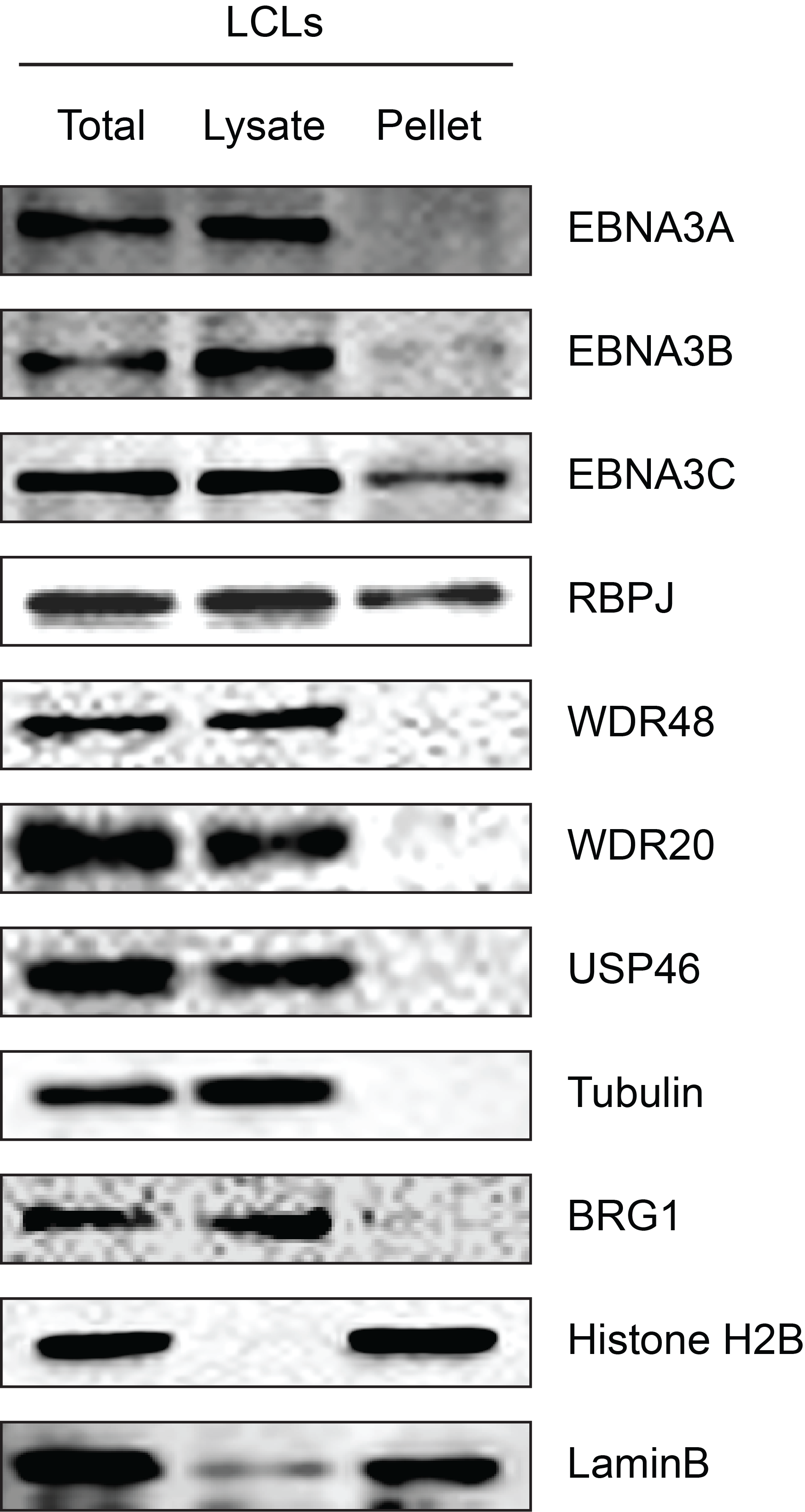

Supplement: S2 Fig — LCLs were lysed in TAP lysis buffer as detailed in the methods section and the residual pellet resuspended as a separate fraction in 1x SDS sample buffer. Total cell lysates, TAP buffer soluble (Lysate) or insoluble (Pellet) fractions were separated by SDS PAGE and probed for EBNA3s, RBPJ, WDR48, WDR20, or USP46 using appropriate antibodies. Fractions were also assessed for control proteins: tubulin (cytoplasm), BRG1 (chromatin associated), Histone H2B (chromatin), and LaminB (cytoskeleton). (TIF) [file ppat.1004822.s002.tif]

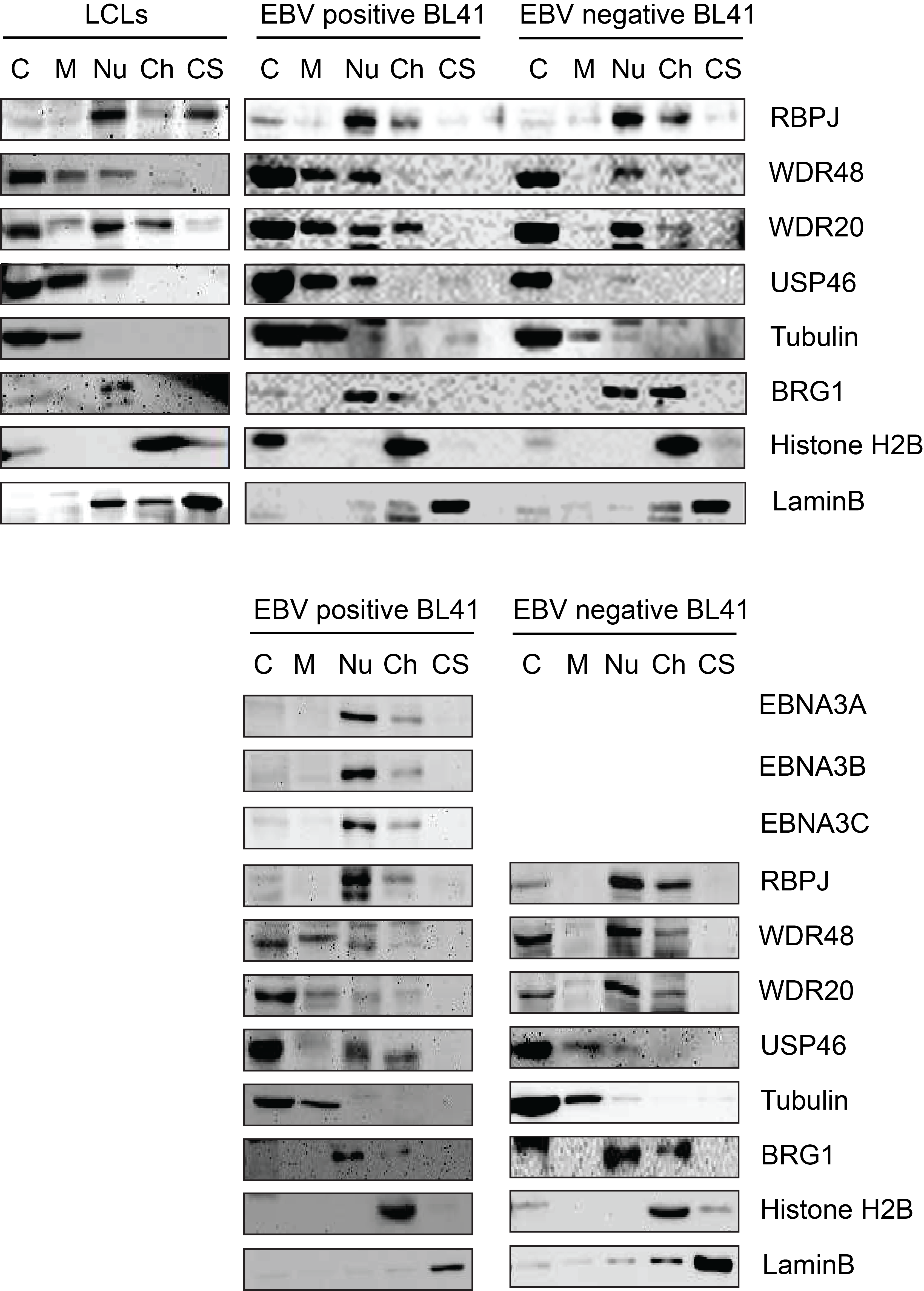

Supplement: S3 Fig — Subcelluar fractionation was performed as for Fig 3 in LCLs, EBV negative BL41 cells and BL41 cells exogenously infected with EBV. Subcellular fractions were blotted with the indicated antibodies. Results of two independent experiments are shown. (TIF) [file ppat.1004822.s003.tif]

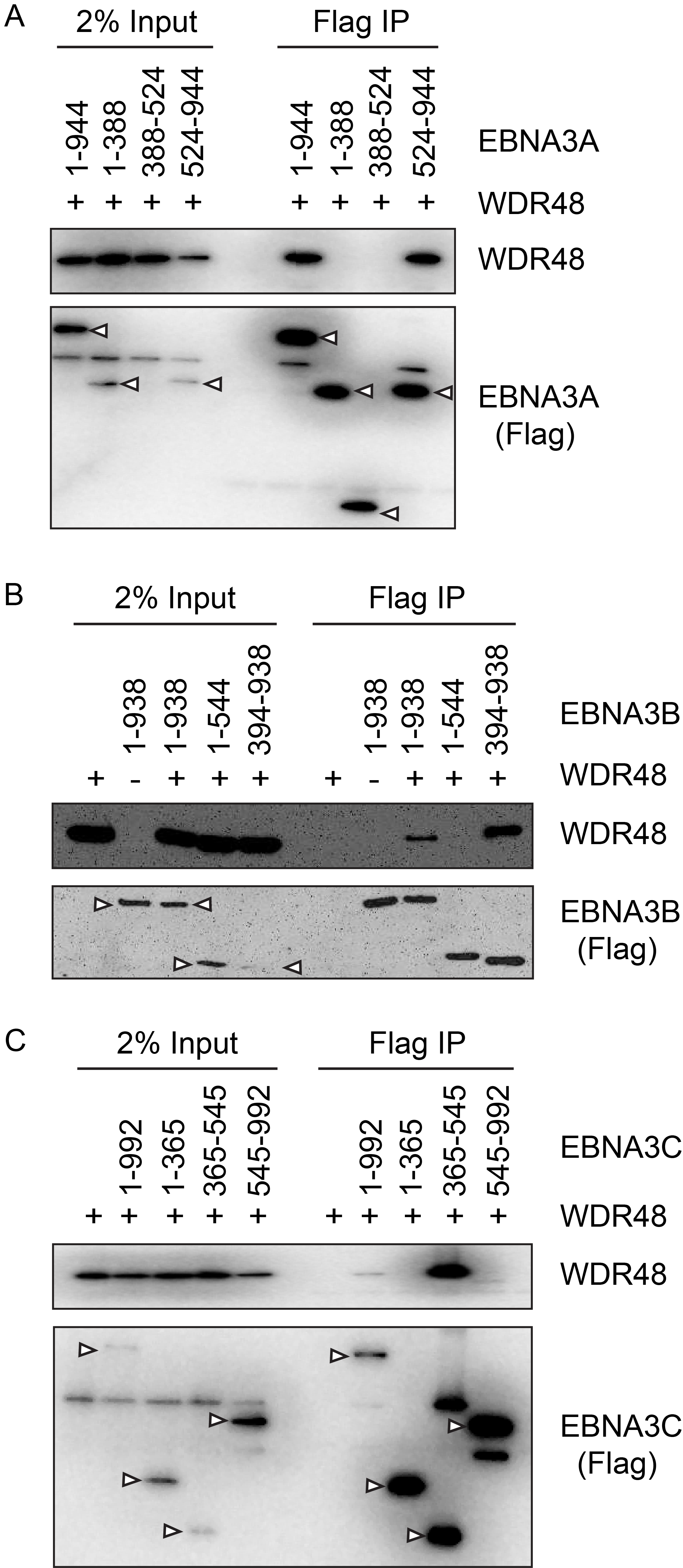

Supplement: S4 Fig — Immunoprecipitation assay to identify WDR48 binding regions with EBNA3A (panel A), EBNA3B (panel B), or EBNA3C (panel C). 293T cells were co-transfected with Xpress tagged WDR48 and flag tagged EBNA3A, EBNA3B, EBNA3C, or the indicated deletion mutants. Lysates were immunoprecipitated with Flag agarose, separated by SDS PAGE, and probed with for Xpress (WDR48) and Flag (EBNA3) antibody as indicated. (TIF) [file ppat.1004822.s004.tif]

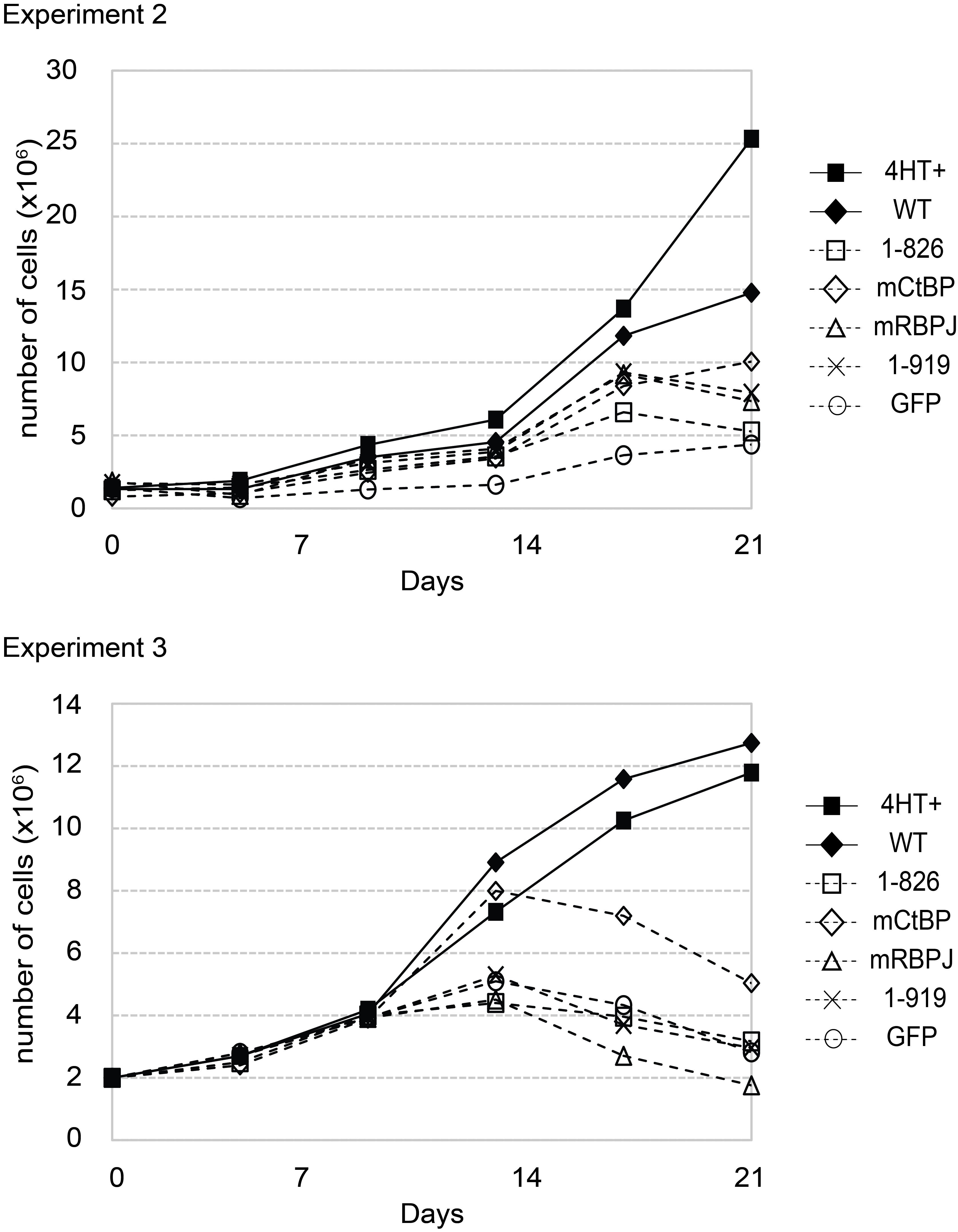

Supplement: S5 Fig — Complementation assays were performed as described in Fig 9 in EBNA3A-HT LCLs. Results of two independent experiments are shown. Growth curves for cells transfected with the following EBNA3A expression plasmids and maintained in the absence of 4HT are shown: EBNA3A WT (closed diamond), EBNA3A 1–826 (open square), EBNA3A mCtBP1 (open diamond), EBNA3A mRBPJ (open triangle), or EBNA3A 1–919 (X) in the absence of 4HT. EBNA3A-HT cells were also transfected with a control GFP expression plasmid, split, and maintained in either the presence (closed square) or absence (open circle) of 4HT. (TIF) [file ppat.1004822.s005.tif]

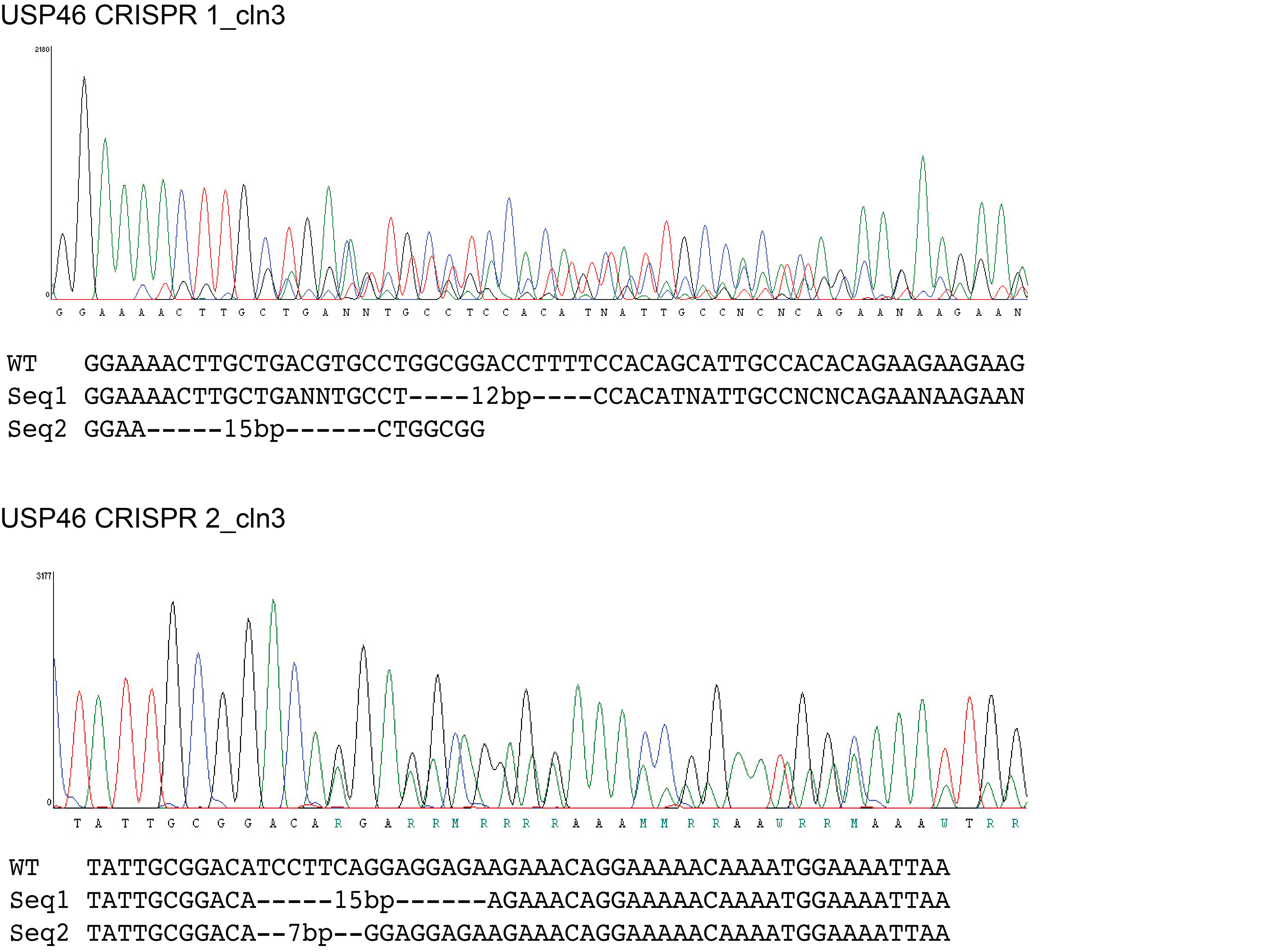

Supplement: S6 Fig — To determine CRISPR-Cas9 editing of the USP46 gene was sucessful in the 721 LCL, we sequenced PCR products from primers flanking the targeted 20mer from one clone for each gRNA. Shown the resultant sequences traces corresponding to 721/ UPS46 CRIPSR1 cln3 and 721/ UPS46 CRIPSR2 cln3 and their interpretations. (TIF) [file ppat.1004822.s006.tif]

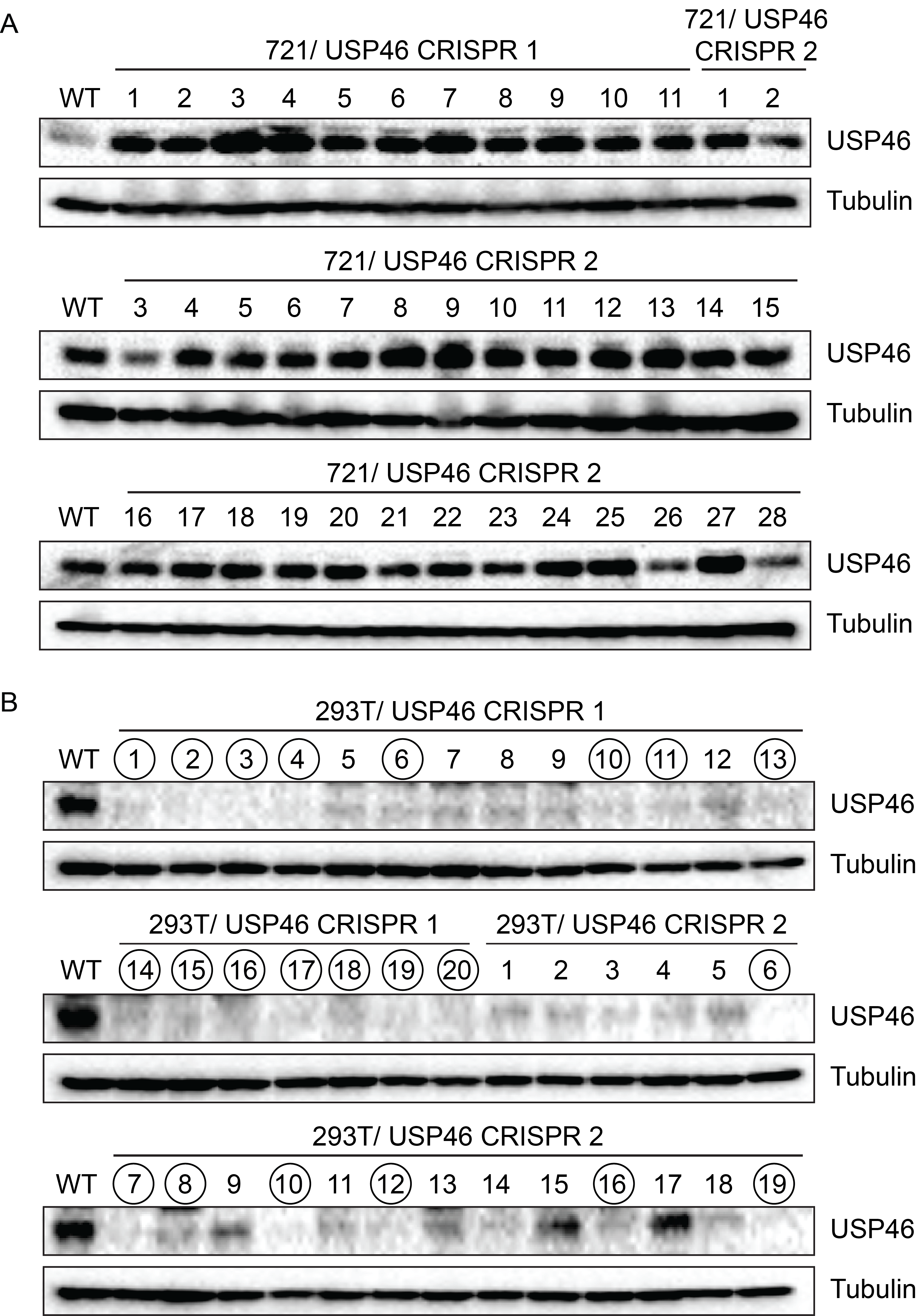

Supplement: S7 Fig — (A) Western blot for USP46 in 721 LCLs cells transfected with a plasmid expressing either of two guide RNAs targeting different USP46 exons as was done in Fig 11. Untransfected 721 cells are also shown (WT). As a loading control, lysates were probed for tubulin (bottom panels). (B) Western blots of untrasnfected 293T cells (WT) or 293T cells that were transfected same CRISPR plasmids and also subjected to one month of hygromycin selection. Cell lines in which USP46 expression was successfully knocked-out are circled. (TIF) [file ppat.1004822.s007.tif]

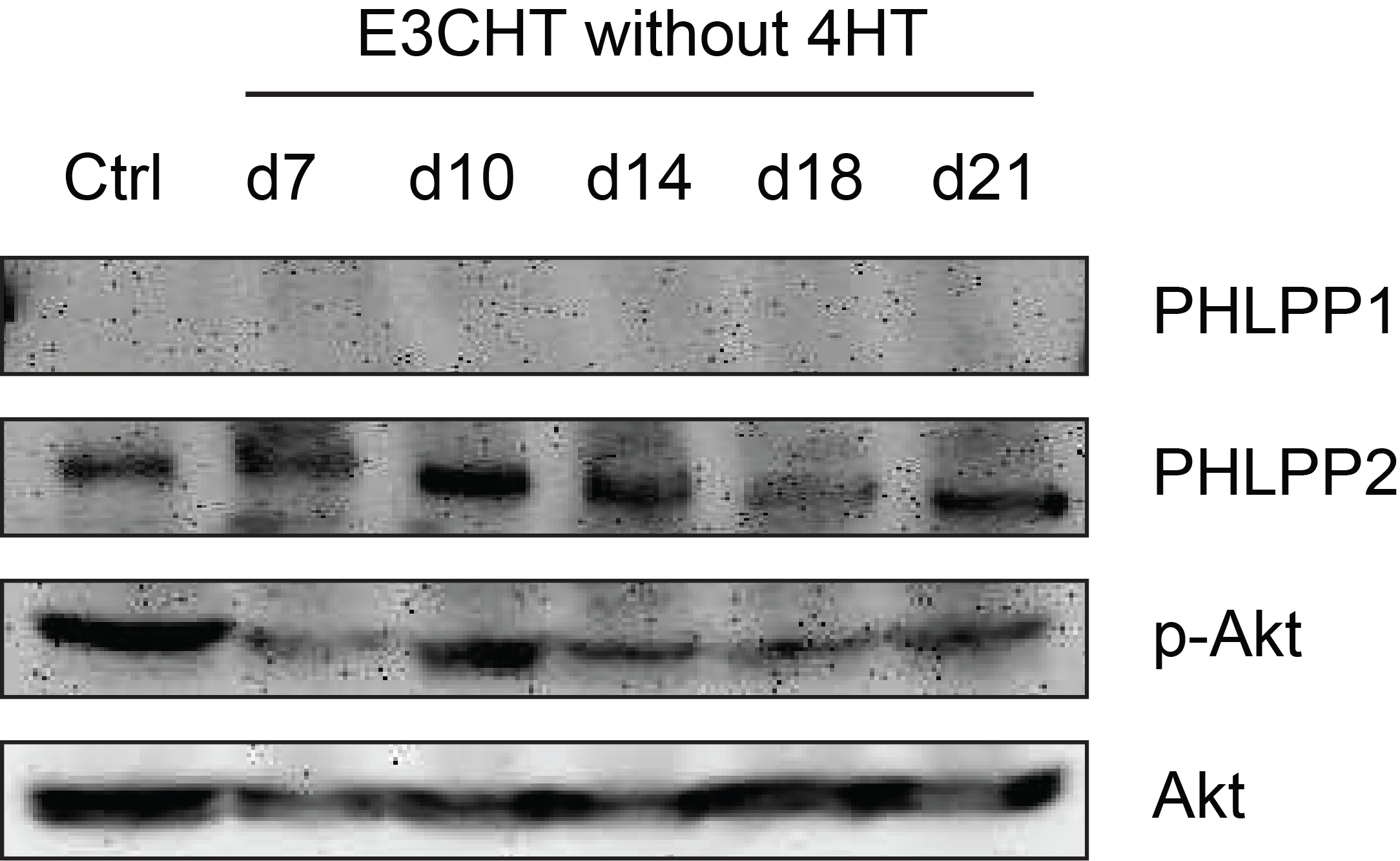

Supplement: S8 Fig — EBNA3C-HT LCLs were grown in the presence of 4HT or harvested at the indicated times after 4HT withdrawal. Levels of PHLPP1, PHLPP2, Akt and phoso-Akt were determined by immunoblotting as indicated. (TIF) [file ppat.1004822.s008.tif]

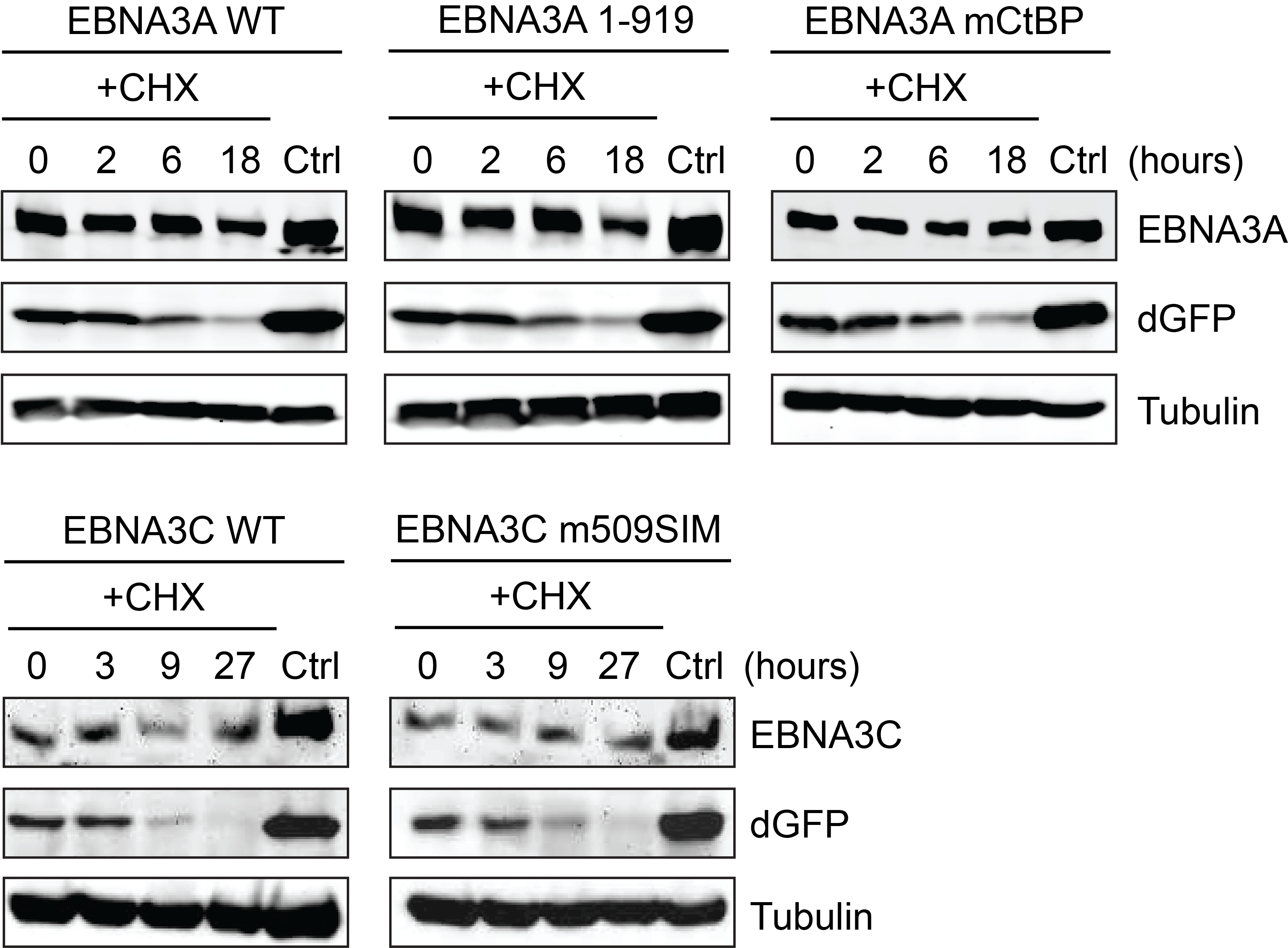

Supplement: S9 Fig — Cells were treated with cycloheximide to determine whether USP46 binding alters EBNA3A or EBNA3C protein stability. EBNA3A wild type, EBNA3A mCtBP1, EBNA3A 1–919 (ΔWDR48), EBNA3C wild type, or EBNA3C 509mSIM was contranfected with destabilized GFP plasmid into 293T cells. After 24 hours cells were treated with 10ug/ml of CHX (0h) and harvested indicated time. Lysates were separated by SDS PAGE and probed with EBNA3A or EBNA3C, GFP, and Tubulin antibodies. (TIF) [file ppat.1004822.s009.tif]

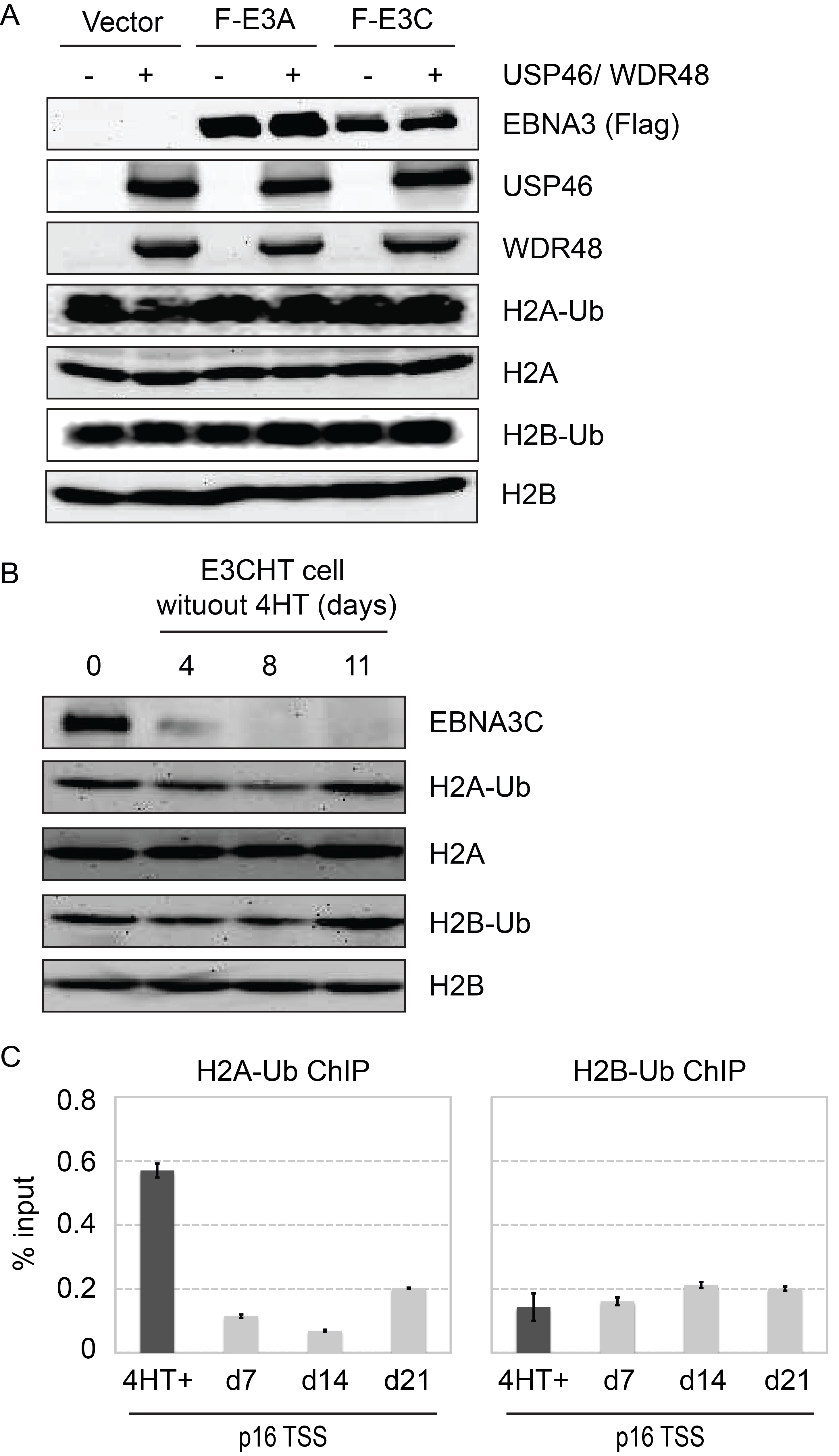

Supplement: S10 Fig — (A) Western blots to detect levels of global H2A and H2B ubiquityaltion in 293T cells transfected with USP46 with or without cotransfected EBNA3A or EBNA3C. (B) Immunoblots demonstrating the effect of inactivation of EBNA3C by 4HT withdrawal in E3C-HT LCLs on global levels of H2A and H2B ubiquitylation. (C) ChIP-assay examining the effect of EBNA3C inactivation in E3C-HT cells on H2A-Ub and U2B-Ub levels at the p16 promoter. This experiment is typical of three independent experiments. (TIF) [file ppat.1004822.s010.tif]
